# Supplementary figures and images for: Association between maternal adversity, DNA methylation, and cardiovascular health of offspring: a longitudinal analysis of the ALSPAC cohort study
Source: BMJ Open. 2022 Mar 23;12(3):e053652. doi: 10.1136/bmjopen-2021-053652 (PMC8948393; doi:10.1136/bmjopen-2021-053652)

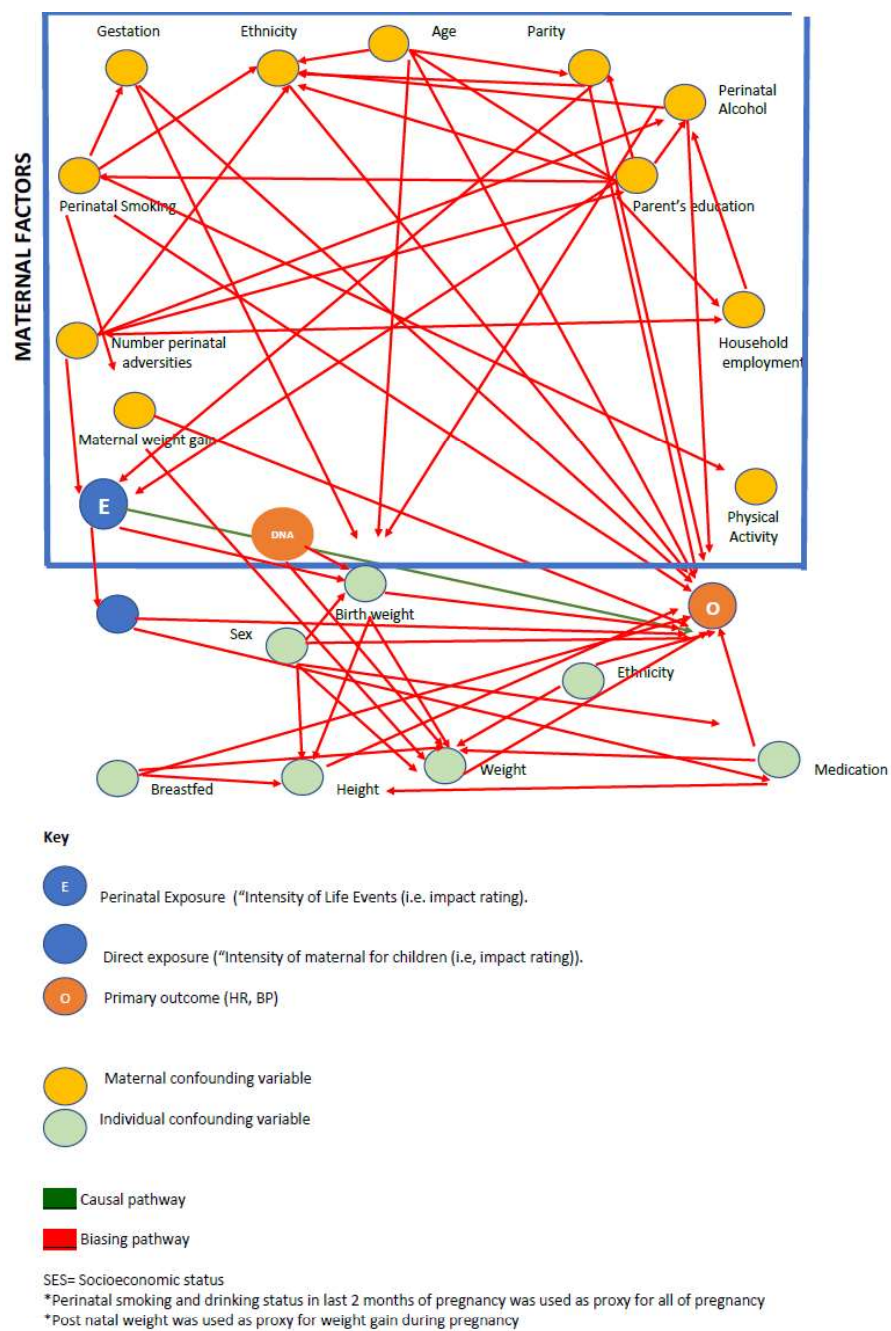

Supplementary Figure S1: Directed Acyclic Graph at 7 years of age

Supplement: Supplementary data [file bmjopen-2021-053652supp001.pdf]

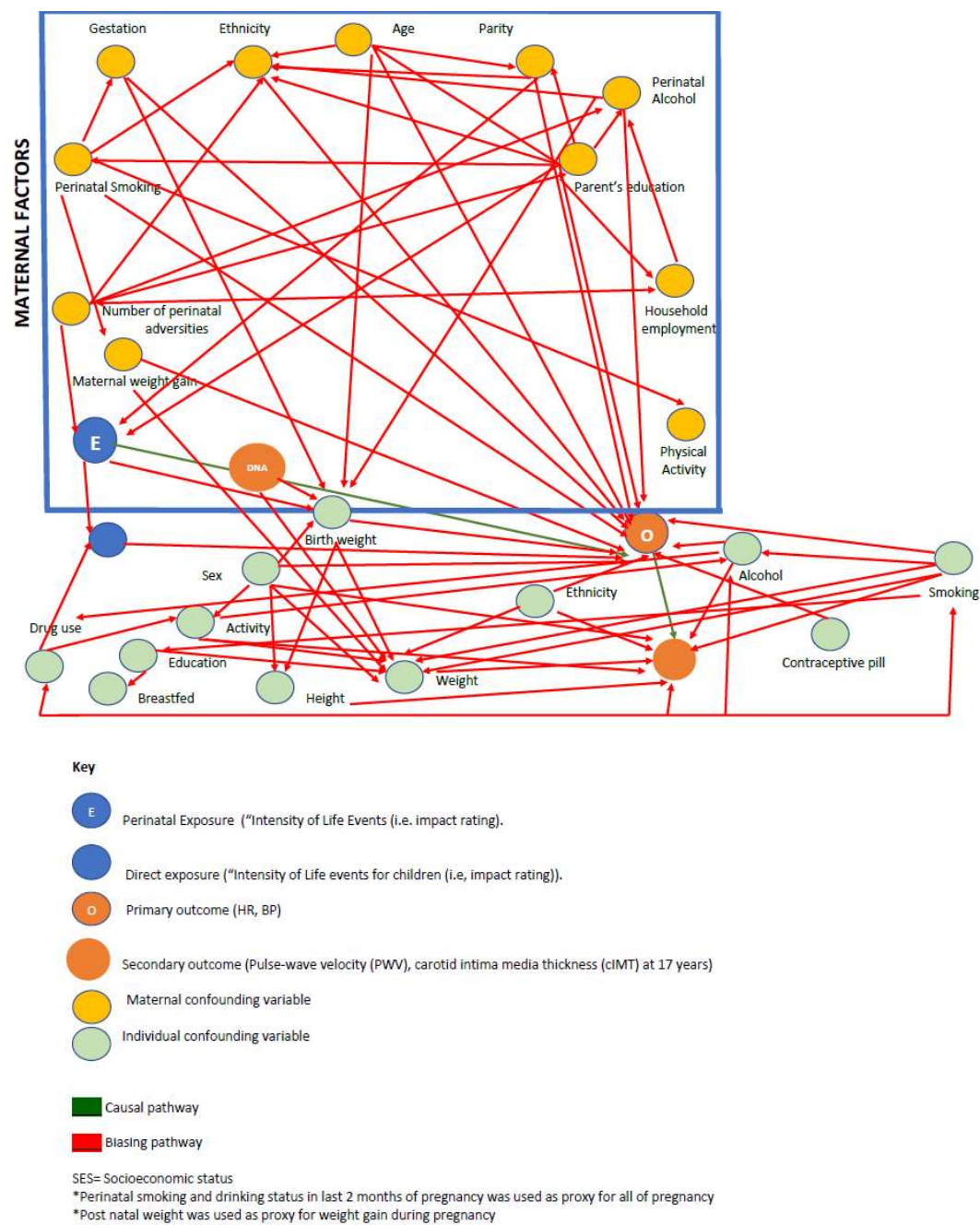

Supplementary Figure S2: Directed Acyclic Graph at seventeen years of age

Supplement: Supplementary data [file bmjopen-2021-053652supp002.pdf]

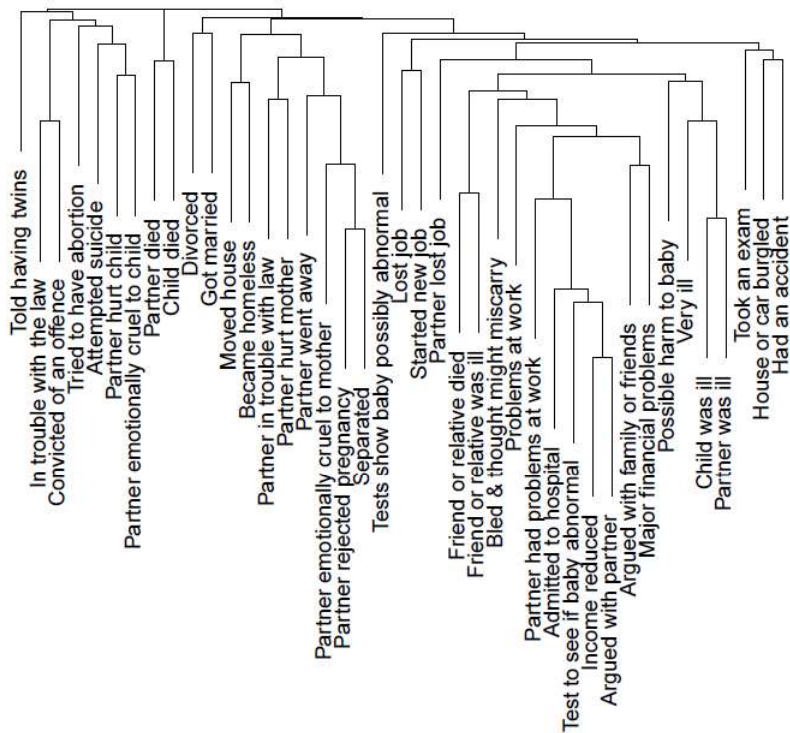

Supplementary Figure S3: Dendrogram of specific life events clusters

Supplement: Supplementary data [file bmjopen-2021-053652supp003.pdf]
