# Supplementary material for: Association between maternal adversity, DNA methylation, and cardiovascular health of offspring: a longitudinal analysis of the ALSPAC cohort study
Source: BMJ Open. 2022 Mar 23;12(3):e053652. doi: 10.1136/bmjopen-2021-053652 (PMC8948393; doi:10.1136/bmjopen-2021-053652)
Supplement: Supplementary data [file bmjopen-2021-053652supp004.pdf]

Supplementary Table S1: Number of people reporting trauma and the perceived impact

Adversity event	n	Occurred n(%)	Did not occur (n%)
Partner died	12240	30 (0.2)	12210 (99.8)
Child died	12245	17 (0.1)	12228 (99.9)
Friend or relative died	12250	2588 (21.1)	9662 (78.9)
Child was ill	12238	2835 (23.2)	9403 (76.8)
Partner was ill	12240	2234 (18.3)	10006 (81.7)
Friend or relative was ill	12244	3182 (26)	9062 (74)
Admitted to hospital	12232	5508 (45)	6724 (55)
In trouble with the law	12246	138 (1.1)	12108 (98.9)
Divorced	12242	157 (1.3)	12085 (98.7)
Partner rejected pregnancy	12234	486 (4)	11748 (96)
Very ill	12244	1393 (11.4)	10851 (88.6)
Partner lost job	12225	1298 (10.6)	10927 (89.4)
Partner had problems at work	12223	3477 (28.4)	8746 (71.6)
Problems at work	12231	1927 (15.8)	10304 (84.2)
Lost job	12231	692 (5.7)	11539 (94.3)
Partner went away	12224	1534 (12.5)	10690 (87.5)
Partner in trouble with law	12228	415 (3.4)	11813 (96.6)
Separated	12232	665 (5.4)	11567 (94.6)
Income reduced	12237	6188 (50.6)	6049 (49.4)
Argued with partner	12243	7727 (63.1)	4516 (36.9)
Argued with family or friends	12243	2965 (24.2)	9278 (75.8)
Moved house	12243	2062 (16.8)	10181 (83.2)
Partner hurt mother	12230	321 (2.6)	11909 (97.4)
Became homeless	12238	239 (2)	11999 (98)
Major financial problems	12237	2319 (19)	9918 (81)
Got married	12236	492 (4)	11744 (96)
Partner hurt child	12229	31 (0.3)	12198 (99.7)
Attempted suicide	12237	28 (0.2)	12209 (99.8)
Convicted of an offence	12231	46 (0.4)	12185 (99.6)
Bled & thought might miscarry	12241	2068 (16.9)	10173 (83.1)
Started new job	12231	628 (5.1)	11603 (94.9)
Test to see if baby abnormal	12233	6430 (52.6)	5803 (47.4)
Tests show baby possibly abnormal	12230	549 (4.5)	11681 (95.5)
Told having twins	12239	113 (0.9)	12126 (99.1)
Possible harm to baby	12239	1287 (10.5)	10952 (89.5)
Tried to have abortion	12241	114 (0.9)	12127 (99.1)
Took an exam	12227	969 (7.9)	11258 (92.1)

Partner emotionally cruel to mother	12220	1100 (9)	11120 (91)
Partner emotionally cruel to child	12222	157 (1.3)	12065 (98.7)
House or car burgled	12239	1221 (10)	11018 (90)
Had an accident	12237	747 (6.1)	11490 (93.9)
