# Supplementary material for: Association between maternal adversity, DNA methylation, and cardiovascular health of offspring: a longitudinal analysis of the ALSPAC cohort study
Source: BMJ Open. 2022 Mar 23;12(3):e053652. doi: 10.1136/bmjopen-2021-053652 (PMC8948393; doi:10.1136/bmjopen-2021-053652)
Supplement: Author's manuscript [file bmjopen-2021-053652.draft_revisions.pdf]

BMJ Open is committed to open peer review. As part of this commitment we make the peer review history of every article we publish publicly available.

When an article is published we post the peer reviewers' comments and the authors' responses online. We also post the versions of the paper that were used during peer review. These are the versions that the peer review comments apply to.

The versions of the paper that follow are the versions that were submitted during the peer review process. They are not the versions of record or the final published versions. They should not be cited or distributed as the published version of this manuscript.

BMJ Open is an open access journal and the full, final, typeset and author-corrected version of record of the manuscript is available on our site with no access controls, subscription charges or pay-per-view fees (http://bmjopen.bmj.com).

If you have any questions on BMJ Open's open peer review process please email info.bmjopen@bmj.com

BMJ Open

# The association between maternal adversity, DNA methylation, and cardiovascular health of offspring


| | |
|---|---|
| Journal: | *BMJ Open* |
| Manuscript ID | bmjopen-2021-053652 |
| Article Type: | Original research |
| Date Submitted by the Author: | 01-Jun-2021 |


| | |
|---|---|
| Complete List of Authors: | Hyde, Natalie; Deakin University, School of Medicine<br>Dowty, James; The University of Melbourne, Centre for Epidemiology and Biostatistics<br>Scovelle, Anna; University of Melbourne VCCC,<br>Armstrong, Gregory; University of Melbourne, Centre for Mental Health, Melbourne School of Population and Global Health<br>Sutherland, Georgina; The University of Melbourne, Centre for Mental Health<br>Olive, Lisa; Deakin, School of Pyschology<br>Lycett, kate; Murdoch Childrens Research Institute, Community Child Health; Deakin, School of Pyschology<br>O'Neil, Adrienne ; Deakin, School of Medicine |


| | |
|---|---|
| Keywords: | CARDIOLOGY, PSYCHIATRY, PERINATOLOGY |
| | |


SCHOLARONE™ Manuscripts

I, the Submitting Author has the right to grant and does grant on behalf of all authors of the Work (as defined in the below author licence), an exclusive licence and/or a non-exclusive licence for contributions from authors who are: i) UK Crown employees; ii) where BMJ has agreed a CC-BY licence shall apply, and/or iii) in accordance with the terms applicable for US Federal Government officers or employees acting as part of their official duties; on a worldwide, perpetual, irrevocable, royalty-free basis to BMJ Publishing Group Ltd ("BMJ") its licensees and where the relevant Journal is co-owned by BMJ to the co-owners of the Journal, to publish the Work in this journal and any other BMJ products and to exploit all rights, as set out in our licence.

The Submitting Author accepts and understands that any supply made under these terms is made by BMJ to the Submitting Author unless you are acting as an employee on behalf of your employer or a postgraduate student of an affiliated institution which is paying any applicable article publishing charge ("APC") for Open Access articles. Where the Submitting Author wishes to make the Work available on an Open Access basis (and intends to pay the relevant APC), the terms of reuse of such Open Access shall be governed by a Creative Commons licence – details of these licences and which Creative Commons licence will apply to this Work are set out in our licence referred to above.

Other than as permitted in any relevant BMJ Author's Self Archiving Policies, I confirm this Work has not been accepted for publication elsewhere, is not being considered for publication elsewhere and does not duplicate material already published. I confirm all authors consent to publication of this Work and authorise the granting of this licence.

For peer review only

# The association between maternal adversity, DNA methylation, and cardiovascular health of offspring

Natalie K Hyde[1*], James G Dowty[2], Anna Scovelle[3], Gregory Armstrong[4], Georgina Sutherland[5], Lisa Olive[6], Kate Lycett[6-8], Adrienne O'Neil[1]

1. Deakin University, IMPACT, the Institute for Mental and Physical Health and Clinical Translation, Barwon Health, School of Medicine, Geelong, Victoria, Australia.
2. Centre for Epidemiology and Biostatistics, The University of Melbourne, Australia
3. Gender & Health Unit, Centre for Health Equity, The University of Melbourne, Australia
4. Nossal Institute of Global Health, The University of Melbourne, Australia
5. Centre for Mental Health, The University of Melbourne, Australia
6. School of Pyschology, Deakin University, Australia
7. Population Health, Murdoch Children's Research Institute, Australia
8. Department of Pediatrics, The University of Melbourne, Australia

***Corresponding author**

Natalie Hyde

Population Health, IMPACT Institute, School of Medicine, Deakin University (Barwon Health), PO Box 281

Geelong, Victoria, Australia, 3220

Email: natalie.hyde@deakin.edu.au

**Word count:**

Manuscript: 3252

**Reference count: 26**



For peer review only - http://bmjopen.bmj.com/site/about/guidelines.xhtml

**Keywords:** adversity, ALSPAC, CV disease, epigenetic, maternal, stress

**Abstract**

**Objectives:** Maternal adversity during pregnancy has been shown to be associated with some offspring health outcomes. This study investigated the association of maternal adversity during pregnancy and DNA methylation with offspring cardiovascular (CV) health.

**Design:** Longitudinal observational cohort study

**Setting:** All pregnant residents in county Avon (~0.9 million) were eligible to participate if their estimated delivery date was between 1 April 1991 and 31 December 1992.

**Participants:** Mother–offspring pairs enrolled in the Avon Longitudinal Study of Parents and Children (ALSPAC) cohort at seven (n=7431) and 17 years of age (n=3143).

**Primary and secondary outcome measures:** Offspring CV health primary measures were heart rate (HR), blood pressure (BP), and secondary meausres were pulse-wave velocity and carotid intima media thickness.

**Results:** Overall, there was no association between maternal adversity scores (number or perceived impact) and primary CV measures. Some small sex effects were observed and there was also a small association between methylation of cg20111643 in cord blood and offspring SBP (1.013-fold change 95% CI: 1.008, 1.017 per standard deviation).

**Conclusions:** We found little evidence to support the overall association of maternal adversity during pregnancy and DNA methylation with offspring CV measures. Sex- and age-specific associations require further investigation.



For peer review only - http://bmjopen.bmj.com/site/about/guidelines.xhtml

**Strengths and limitations of this study**

- A strength of this study is longitudinal collection of phenotypic data in both women and their child.
- A limitation is attrition bias, with those of a higher socioeconic status being more likely to remain in the study over time.
- In addition it is possible that some life stressors during pregnancy may have not have been captured.

For peer review only



For peer review only - http://bmjopen.bmj.com/site/about/guidelines.xhtml

## Introduction

Seminal work conducted by Barker et al. in the early twentieth century noted geographical differences in infant mortality rates, whereby regions of England with the highest infant mortality rates also had the highest rate of coronary heart disease (CHD) mortality[1 2]. From this, it was concluded "*...adverse environmental influences in utero and during infancy, associated with poor living standards, directly increased susceptibility to the disease (CHD).*" Further extending this work, the Developmental Origins of Health and Disease (DOHaD) hypothesis[3] proposes that the risk of chronic disease, such as cardiovascular disease (CVD), originate not only from an individual's genome but also by its interactions with biological insults *in utero* and early life.

To date, much of the work in this area has focussed on the impact of maternal nutrition during pregnancy, with comparatively fewer data on social adversity and trauma. However, there are some data to suggest that the ways in which women experience social adversity during pregnancy may induce similar changes to disease trajectory in the offspring as maternal malnourishment[4].

The time *in utero* represents a critical period of development, which may be particularly vulnerable to maternal stress. During this time, the fetus is directly susceptible to the biological effects of maternal stress owing to its reliance on the maternal blood supply via the placenta. Epigenetics are mitotically heritable changes to gene expression that do not involve changes to the underlying genetic sequence. These changes in gene expression may provide some clues about the mechanisms through which maternal adversity embeds itself into an individual and her offspring. A recent review of maternal prenatal stress and infant DNA methylation



For peer review only - http://bmjopen.bmj.com/site/about/guidelines.xhtml

identified several candidate genes implicated in the maternal central stress response that may be critical in driving phenotype changes for offspring[4].

The extent of cumulative damage to biological systems that occurs with increasing number, duration or severity of exposures, particularly with age, is likely to be a critical consideration in understanding associations between maternal adversity and the cardiovascular (CV) health of a child. This includes distinguishing the response (e.g., perceived stress) from the stimulus or stressor (e.g., the adversity) itself. It is also notable that there appears to be a sexually dimorphic response with regards to several developmental exposures and cardiovascular conditions[5]. These issues, along with other key gaps in the evidence base that exist in psycho-cardiology have been outlined in the position paper by the American Heart Association (AHA)[6]. Specifically, these gaps relate to, (i) an absence of truly prospective studies that commenced in the pre-natal period with capacity to explicate this relationship, and (ii) a lack of studies that identify the biological mechanisms linking adversity to CVD.

This study therefore seeks to, (i) investigate the respective and cumulative impact of women's exposure(s) to adversity during the perinatal period, and cord blood DNA methylation on CV health of her offspring, (ii) establish whether associations are sex or age-dependent, and (iii) determine whether DNA methylation at birth is associated with CV outcomes. We hypothesise that greater maternal adversity will be associated with poorer CV health of offspring and DNA methylation will be associated with offspring CV measures.



For peer review only - http://bmjopen.bmj.com/site/about/guidelines.xhtml

## Methods

### Study design and participants

This study used longitudinal data from the Avon Longitudinal Study of Parents and Children (ALSPAC formerly "Children of the 90s" study). ALSPAC is a prospective birth cohort study conducted in the United Kingdom. The full study protocol is available elsewhere with participation rates and reason for not participation [7 8]. Briefly, all pregnant female residents in county Avon (~0.9 million) were eligible to participate if their estimated delivery date was between 1 April 1991 and 31 December 1992 inclusive. Recruitment occurred via maternity health services and mass media campaigns. After their initial expression of interest and assessment of eligibility by ALSPAC staff, women were sent the baseline questionnaire ~1 week later. The women of 14,541 pregnancies (71.8% of all pregnancies in the area at that time) were recruited antenatally during 1990–92. They completed a series of postal questionnaires throughout their pregnancy and there were several clinical assessments post-birth. CV health data were collected when the children were aged seven and 17 years.

There were 13,617 mother-offspring pairs from singleton live births who survived to ≥1 year of age; only singleton pregnancies and those women with term deliveries were included in the analyses. When the oldest children were approximately 7 years of age, an attempt was made to bolster the initial sample with eligible cases who had failed to join the study originally, resulting in an additional 913 children being enrolled. The total sample size for analyses using any data collected after the age of seven is therefore 15,454 pregnancies, resulting in 15,589 foetuses. Of these 14,901 were alive at 1 year of age. The number of children with CV measures at the subsequent 7- and 17-year time points were 7431 and 5215, respectively and were included in analyses if they had complete information for the relevant analyses.

### Patient and public involvement



For peer review only - http://bmjopen.bmj.com/site/about/guidelines.xhtml

There was no direct involvement from participants in the study design. Select participants are part of a committee which meets to discuss and provide insights on acceptability, and study methodology and design.

**Measures**

*Exposure variable*. Data were those provided by women at (i) 0-18 weeks gestation, and (ii) 19-weeks gestation and 8-weeks postpartum. Women retrospectively self-reported social adversities and rated its impact for the respective period. Adverse life events were assessed using a 41-item self-report questionnaire based on a Life Events Inventory[9], using the average score at the two timepoints. The internal reliability of the inventory, as indicated by the coefficient, is 0.68. Each item was rated in one of five categories: "Yes, affected me a lot," "Yes, affected me moderately," "Yes, affected me mildly," "Yes, but did not affect me," and "No, did not happen" and was rated from 0 to 4, with higher scores indicating greater perceived stress. Two scores were calculated as follows: (1) the number of stressful life and (2) the perceived impact of the events.

*Outcome variables*. The primary outcomes were blood pressure and heart rate at 7 and 17 years of age. Duplicate measures of resting heart rate, systolic BP (SBP) and diastolic BP (DBP) were taken using a Dinamap 9301 Vital Signs Monitor whilst participants were seated, using the average of the two readings.

Secondary outcomes were pulse-wave velocity (PWV) and carotid intima media thickness (cIMT) at 17 years of age, using the mean of three measures and the mean of three end-diastolic measurements of both the left and right side, respectively. A Vicorder device (Skidmore Medical, UK) was used to measure PWV and a Zonare Z.OneUltra system that had a a L10-5



For peer review only - http://bmjopen.bmj.com/site/about/guidelines.xhtml

linear transducer (Zonare Medical Systems, CA, US) was used to determine cIMT. Detailed protocols have been described elsewhere[10].

*DNA methylation data.* Embedded within the ALSPAC study is a human epigenetic resource; the Accessible Resource for Integrated Epigenomic Studies (ARIES)[11]. Of the 1018 mother–offspring pairs in the ARIES project, 916 offspring had cord blood methylation data, which passed quality control [12]. Cord blood at birth was used to assess epigenome-wide methylation levels using the Illumina Infinium® HumanMethylation450 (HM450) BeadChip.

Raw intensity signals were processed and M-values were calculated using the minfi package [13]. Probes and samples were removed if they failed quality assurance based on their detection p-values. All samples were Illumina and SWAN normalised to reduce technical bias between Type 1 and Type 2 probes.

*Confounding variables.* Directed acyclic graphs (DAGs) were constructed (Supplementary Figures S1 and S2) from which a minimal set of adjusted variables were selected using the R packages ggdag and dagitty. In the primary analyses, the final models were adjusted for child age, alcohol use in pregnancy, tobacco use in pregnancy, ethnic group, parity, age at delivery, and maternal education. All methylation analyses were additionally adjusted for white blood cell composition, using the algorithm by Houseman et al.[14].

Please note that the study website contains details of all the data that is available through a fully searchable data dictionary and variable search tool" and reference the following webpage: http://www.bristol.ac.uk/alspac/researchers/our-data/. Ethical approval for the study was obtained from the ALSPAC Ethics and Law Committee and the Local Research Ethics Committees and The University of Melbourne Human Research Ethics committee (ref: 1853268.1). Consent for biological samples has been collected in accordance with the Human



For peer review only - http://bmjopen.bmj.com/site/about/guidelines.xhtml

Tissue Act (2004) and informed consent for the use of data collected via questionnaires and clinics was obtained from participants following the recommendations of the ALSPAC Ethics and Law Committee at the time.

**Statistical analyses**

Outcomes were log-transformed for anaylses. Linear mixed models[15] with random intercepts (one for each offspring) were used to analyse the association between these longitudinal outcome variables and various exposure variables (individual adverse events, the number of such events, the perceived impact of such events, and methylation variables). Sub-analyses were also conducted to estimate the association between the exposures and the log-transformed CV measures at each age separately, and linear regression was used for these analyses instead of linear mixed models and adjusted for the minimal set of potential confounders. All estimates of associations for CV measures are for a 4-unit change in maternal adversities, which corresponds to the difference between the adversity not occurring and the adversity having its highest impact. Linear mixed models[15] were used to test the associations between individual CpG sites with maternal adversity measures and child CV measures. The Bonferroni p-value threshold was used to correct for multiple testing in the analyses of individual methylation probes.

P-values were based on the likelihood ratio statistic except for the descriptive analyses, where p-values for a sex difference were based on a t-test (for continuous variables) or Fisher's exact test (for binary variables)[16]. All analyses were conducted in R version 4.0.0[17].



For peer review only - http://bmjopen.bmj.com/site/about/guidelines.xhtml

## Results

Characteristics and summary data of the sample are as shown in Table 1. The median number of maternal events and perceived impact score was 3.6 (2.3) and 8.5 (7), respectively (Table 1). The most common event during the study period was an argument with partner (63.1%), followed by foetal testing (52.6%) and reductions in income (50.6%) (Supplementary Table S1).

### Maternal adversity and overall offspring CV measures

There was no association between number of events and any of the primary offspring CV measures (CV time points combined) (Table 2). Results did not differ when analyses were re-run using perceived impact scores.

### Maternal adversity and offspring CV measures by sex and specific timepoints

In contrast to our hypotheses, there was an association between perceived impact score and PWV (time points combined) in boys, whereby a four-unit increase in adversity score was associated with a 0.1% decrease in PWV (0.999-fold change, 95% CI: 0.997, 1.001; Table 2). When HR and BP measures were examined at specific time points (i.e., 7-yrs and 17-yrs separately) there was an association between maternal number of events and offspring SBP at seven years of age in girls, whereby there was a 0.6% decrease in BP for each additional four events (0.994-fold change, 95% CI: 0.988, 0.999). In line with our hypotheses, there was also an association between offspring DBP at 17-years of age and maternal perceived impact score in girls whereby a four-unit increase in impact score was associated with a 0.2% increase in DBP (1.002-fold change 95% CI: 1.000, 1.005). There were no other associations detected with number of events or perceived impact score at specific time points (data not shown).



For peer review only - http://bmjopen.bmj.com/site/about/guidelines.xhtml

### DNA methylation and offspring CV measures

In line with our hypotheses, there were some associations evident with specific CpG sites. In the longitudinal analyses, with timepoints combined, methylation of cg20111643 was associated with offspring SBP (1.013-fold change 95% CI: 1.008, 1.017 per standard deviation). There was an association with methylation of cg07494499 (1.012-fold change 95% CI: 1.008, 1.017 per standard deviation of the outcome) and cg02458152 (1.011-fold change 95% CI: 1.007, 1.015 per standard deviation) and SBP. There was also an association between methylation of cg20222926 (0.987-fold change 95% CI: 0.982, 0.992 per standard deviation) and DBP that appeared to be largely driven by rare, large DNA methylation changes (Figure 1). However, when the 3 outliers were excluded, the effect was no longer observed. There were no associations with any other CpG site.

### Specific adversities and offspring CV measures

We further explored how events clustered (Supplementary Figure S3), and whether specific events were associated with offspring CV measures stratified by sex (Table 3).

Of the 43 maternal adversity events examined, 5 showed associations with slightly favourable CV health in the offspring at age 7 years. These associations differed by sex.

In contrast to the hypotheses, at the seven-year follow-up, mothers who had argued with their partner during pregnancy had female offspring with 2.4% lower HR (0.976 fold-change 95% CI: 0.961, 0.992); those who took an exam during pregnancy had female offspring with 4.2% lower SBP (0.958-fold-change 95% CI: 0.928, 0.989); and those admitted to hospital during pregnancy had female offspring with 2.5% lower DBP (0.975 fold-change 95% CI: 0.956, 0.992). Mothers who had an ill partner during pregnancy had male offspring with 2.9% lower SBP (0.971-fold change 95% CI: 0.951, 0.992); and those who become homeless had male offspring with 7.3% lower DBP (0.927-fold change 95% CI: 0.88, 0.978). However, in



For peer review only - http://bmjopen.bmj.com/site/about/guidelines.xhtml

agreement with the hypotheses, at 17-years, mothers whose partner rejected the pregnancy had female offspring with 7.2% higher HR (1.072-fold change 95% CI: 1.019, 1.127); and mothers who moved house during pregnancy had male offspring with 4.5% higher HR (1.045-fold change 95% CI: 1.012, 1.079). Mothers who were convicted of an offence or took an exam during pregnancy had male offspring with 91.9% (1.919-fold change 95% CI:1.209, 3.041) and 9.8% (1.098-fold change 95% CI: 1.027, 1.174) higher HR. Women who reported they were very ill during pregnancy had male offspring with 3.2% higher SBP (1.032-fold change 95%CI: 1.009, 1.056); and 4.2% DBP (1.042-fold change 95% CI: 1.03, 1.072), respectively. Contrasting the hypotheses, at 17 years of age mothers whose partner hurt the child during pregnancy had male offspring with 34.5% lower HR (0.655-fold change 95% CI: 0.492, 0.873).

For peer review only

**Discussion**

There was no evidence of an overall association between our primary CV measures in offspring and maternal adversity. There was limited evidence to suggest that sub-types of adversity or specific CpG probes may be associated with CV measures in an age-specific manner.

Associations between adversity and health outcomes previously reported in the literature are thought to be moderated by biological changes induced by the stress response. Global methylation is associated with CVD in adult populations[18]. However, the association between epigenetic changes at birth and CV measures in childhood and adolescence is less well-characterised. It could be that infancy and childhood is a more sensitive period to CV changes induced by adversity than during pregnancy and, although previous results are mixed. For instance,there is support for associations between childhood maltreatment and CV disease and risk factors in adulthood[19]. It is possible that exposure to adversities experienced by this cohort were not severe, nor prolonged enough, to have a direct impact on DNA methylation and/or on cardiac function. There were some associations with specific CpG sites, cg20111643 (TOM1L1), cg07494499 (NXN), cg02458152 and cg20222926 (FEZF1). Of the genes that these sites are located on only one, NXN, has a postulated role in cardiac development through its role in the canonical Wnt/β-catenin signalling pathway[20]. Of note is the association with cg20222926, which may be the result of interesting biology, or could be a consequence of measurement error.

Few studies have looked at maternal adversity and CV risk factors in childhood and adolescence. Within this cohort, no association was observed between childhood adversity and blood pressure at seven and 11 years of age[21]. In an Australian cohort of children those with lower psychosocial stress had higher pulse pressure at age 11[22]. This finding is similar to the



For peer review only - http://bmjopen.bmj.com/site/about/guidelines.xhtml

favourable associations observed in our study between specific adversities and offspring CV measures, at 7 years of age. Given that this is a paediatric study population it is possible that the unexpected increases in BP observed at seven years of age may be a feature of the developing CV system in the offspring[23]. Of further consideration is that CV measures during childhood and adolescence may not wholly predict progression to CV disease in adulthood[24]. Thus, the results presented do not preclude further examination of perinatal adversity and CV disease and risk in adulthood. It is plausible that the risk pathways between maternal stress and CVD risk are activated, but the damage is not yet evident. This would be consistent with the accumulation hypothesis of lifecourse epidemiology, which purports that health disparities become more pronounced with age (i.e diverge)[25]. Moreover, the measure of maternal adversity used in this study was an inventory of life events, not based on a conceptual framework, such as that of the adverse childhood experiences construct. Thus, this measure of adversity may not have captured all stressors during pregnancy, which may conceal a legitimate association and in part explain the null findings.

Specific adversities were associated with favourable changes in offspring CV measures at age seven. At age 17, the direction of the association largely reversed. This is suggestive of a protective adaptive response to maternal adversity present in childhood that may reverse trajectory by age 17. Contrary to the original hypotheses, at age seven, specific maternal adversities largely appeared to have a protective effect on offspring CV measures. Similarly, in this same cohort, a different study observed that maternal prenatal anxiety and depressive symptomology was inversely associated with offspring blood pressure at 10-11 years of age, albeit to a similar magnitude as paternal measures[26]. However, given this association was not looked at beyond 11 years of age it is not known if a similar reversal of trajectory was present at 17-years. Given multiple comparisons, it is also possible that the associations between



For peer review only - http://bmjopen.bmj.com/site/about/guidelines.xhtml

specific adversities and offspring CV have arisen due to chance. However, it is curious that the associations largely follow the same age-trajectory, that is an inverse association with adversity events at seven years and a positive association at 17 years. It is also noteworthy that reported adversities that had the largest effect size were those that would presumably have more psychological impact e.g. partner hurt child and mother convicted of an offence. Nevertheless, replication in other cohorts would have to be demonstrated to confirm such associations.

A strength of the current study is its large sample size and its detailed collection of longitudinal phenotypic data in both mothers and their children followed into adolescence. However, as is the case with such long-term observational studies, over time, there is evidence of attrition, which may introduce bias, with those who were of a higher socioeconomic position being more likely to remain in the study over time thus potentially limiting the generalisabilty of the results. Moreover, the list of potentially life stressors was not exhaustive and may have resulted in measurement error influencing the results. In addition, the adversity scores calculated as part of this study have not been previously validated. Furthermore, to capture maternal adversity during pregnancy, we took the weighted average of the Life Events Inventory, which was inclusive from the beginning of pregnancy to 8 weeks post-partum). Thus, any effects may have been diluted by the inclusion of adversity in the eight weeks post birth during the perinatal period.

In summary, the results presented largely do not support an association between maternal prenatal adversity, and offspring methylation and CV measures during childhood and adolescence. There were, however some sex- and age- specific trends which would have to be confirmed in future studies.

For peer review only

**Funding statement**

The UK Medical Research Council and Wellcome (Grant ref: 217065/Z/19/Z) and the University of Bristol provide core support for ALSPAC. A comprehensive list of grants funding is available on the ALSPAC website (http://www.bristol.ac.uk/alspac/external/documents/grant-acknowledgements.pdf); This research was specifically funded by the British Heart Foundation who provided support for the collection of pulse wave velocity measures (RG/10/004/28240GWAS). Data was generated by Sample Logistics and Genotyping Facilities at Wellcome Sanger Institute and LabCorp (Laboratory Corporation of America) using support from 23andMe. This specific analyses were supported by a grant from the University of Melbourne.

**Competing interests statement**

None declared.

**Authorship statement**

AO conceived the initial idea for examining the associations and all authors made a substantial contribution to the conception and/or design of the study analyses. JGD performed all statistical analyses. NKH wrote the initial draft of the manuscript and all authors reviewed and contributed intellectual content. All authors have approved of the final version that has been submitted.

For peer review only

## References

1. Barker DJ, Osmond C, Law CM. The intrauterine and early postnatal origins of cardiovascular disease and chronic bronchitis. *Journal of Epidemiology and Community Health* 1989;43(3):237-40. doi: 10.1136/jech.43.3.237
2. Barker DJ, Osmond C, Golding J, et al. Growth in utero, blood pressure in childhood and adult life, and mortality from cardiovascular disease. *British Medical Journal* 1989;298(6673):564-67. doi: 10.1136/bmj.298.6673.564
3. Gillman MW, Barker D, Bier D, et al. Meeting report on the 3rd International Congress on Developmental Origins of Health and Disease (DOHaD). *Pediatric research* 2007;61(5 Pt 1):625-9. doi: 10.1203/pdr.0b013e3180459fcd [published Online First: 2007/04/07]
4. Sosnowski DW, Booth C, York TP, et al. Maternal prenatal stress and infant DNA methylation: A systematic review. *Developmental psychobiology* 2018;60(2):127-39. doi: 10.1002/dev.21604 [published Online First: 2018/01/19]
5. Gilbert JS. Chapter 8 - Epigenetics in the Developmental Origin of Cardiovascular Disorders. In: Rosenfeld CS, ed. The Epigenome and Developmental Origins of Health and Disease. Boston: Academic Press 2016:127-41.
6. Suglia SF, Koenen KC, Boynton-Jarrett R, et al. Childhood and Adolescent Adversity and Cardiometabolic Outcomes: A Scientific Statement From the American Heart Association. *Circulation* 2018;137(5):e15-e28. doi: 10.1161/cir.0000000000000536 [published Online First: 2017/12/20]
7. Boyd A, Golding J, Macleod J, et al. Cohort Profile: The 'Children of the 90s'—the index offspring of the Avon Longitudinal Study of Parents and Children. *International Journal of Epidemiology* 2012;42(1):111-27. doi: 10.1093/ije/dys064
8. Fraser A, Macdonald-Wallis C, Tilling K, et al. Cohort Profile: The Avon Longitudinal Study of Parents and Children: ALSPAC mothers cohort. *International Journal of Epidemiology* 2012;42(1):97-110. doi: 10.1093/ije/dys066
9. Barnett BE, Hanna B, Parker G. Life event scales for obstetric groups. *Journal of psychosomatic research* 1983;27(4):313-20. doi: 10.1016/0022-3999(83)90054-5 [published Online First: 1983/01/01]
10. O'Keeffe LM, Howe LD, Fraser A, et al. Associations of Y chromosomal haplogroups with cardiometabolic risk factors and subclinical vascular measures in males during childhood and adolescence. *Atherosclerosis* 2018;274:94-103. doi: 10.1016/j.atherosclerosis.2018.04.027 [published Online First: 2018/05/13]
11. Relton CL, Gaunt T, McArdle W, et al. Data Resource Profile: Accessible Resource for Integrated Epigenomic Studies (ARIES). *Int J Epidemiol* 2015;44(4):1181-90. doi: 10.1093/ije/dyv072 [published Online First: 2015/05/21]
12. Richmond RC, Simpkin AJ, Woodward G, et al. Prenatal exposure to maternal smoking and offspring DNA methylation across the lifecourse: findings from the Avon Longitudinal Study of Parents and Children (ALSPAC). *Human molecular genetics* 2015;24(8):2201-17. doi: 10.1093/hmg/ddu739 [published Online First: 2015/01/02]
13. Aryee MJ, Jaffe AE, Corrada-Bravo H, et al. Minfi: a flexible and comprehensive Bioconductor package for the analysis of Infinium DNA methylation microarrays. *Bioinformatics (Oxford, England)* 2014;30(10):1363-9. doi: 10.1093/bioinformatics/btu049 [published Online First: 2014/01/31]
14. Houseman EA, Accomando WP, Koestler DC, et al. DNA methylation arrays as surrogate measures of cell mixture distribution. *BMC Bioinformatics* 2012;13(1):86. doi: 10.1186/1471-2105-13-86
15. Schall R. Estimation in Generalized Linear Models with Random Effects. *Biometrika* 1991;78(4):719-27. doi: 10.2307/2336923
16. Agresti A. Categorical data analysis: John Wiley & Sons 2003.



For peer review only - http://bmjopen.bmj.com/site/about/guidelines.xhtml

17. Team RC. R: A language and environment for statistical computing: Vienna, Austria, 2013.
18. Muka T, Koromani F, Portilla E, et al. The role of epigenetic modifications in cardiovascular disease: A systematic review. *International journal of cardiology* 2016;212:174-83. doi: 10.1016/j.ijcard.2016.03.062 [published Online First: 2016/04/04]
19. Basu A, McLaughlin KA, Misra S, et al. Childhood Maltreatment and Health Impact: The Examples of Cardiovascular Disease and Type 2 Diabetes Mellitus in Adults. *Clinical psychology : a publication of the Division of Clinical Psychology of the American Psychological Association* 2017;24(2):125-39. doi: 10.1111/cpsp.12191 [published Online First: 2017/09/05]
20. Mak CCY, Chow PC, Liu APY, et al. De novo large rare copy-number variations contribute to conotruncal heart disease in Chinese patients. *npj Genomic Medicine* 2016;1(1):16033. doi: 10.1038/npjgenmed.2016.33
21. Slopen N, Koenen KC, Kubzansky LD. Cumulative adversity in childhood and emergent risk factors for long-term health. *The Journal of pediatrics* 2014;164(3):631-8.e1-2. doi: 10.1016/j.jpeds.2013.11.003 [published Online First: 2013/12/19]
22. Olive LS, Abhayaratna WP, Byrne D, et al. Depression, stress and vascular function from childhood to adolescence: A longitudinal investigation. *General Hospital Psychiatry* 2020;62:6-12. doi: https://doi.org/10.1016/j.genhosppsych.2019.10.001
23. Wilkinson IB, Franklin SS, Hall IR, et al. Pressure amplification explains why pulse pressure is unrelated to risk in young subjects. *Hypertension (Dallas, Tex : 1979)* 2001;38(6):1461-6. doi: 10.1161/hy1201.097723 [published Online First: 2001/12/26]
24. Urbina EM, Khoury PR, Bazzano L, et al. Relation of Blood Pressure in Childhood to Self-Reported Hypertension in Adulthood. *Hypertension (Dallas, Tex : 1979)* 2019;73(6):1224-30. doi: 10.1161/hypertensionaha.118.12334 [published Online First: 2019/05/09]
25. Dannefer D. Cumulative Advantage/Disadvantage and the Life Course: Cross-Fertilizing Age and Social Science Theory. *The Journals of Gerontology: Series B* 2003;58(6):S327-S37. doi: 10.1093/geronb/58.6.S327
26. van Dijk AE, Dawe K, Deanfield J, et al. The association of maternal prenatal psychosocial stress with vascular function in the child at age 10-11 years: findings from the Avon longitudinal study of parents and children. *Eur J Prev Cardiol* 2014;21(9):1097-108. doi: 10.1177/2047487313486039 [published Online First: 2013/04/04]

For peer review only

**Table 1:** Participant characteristics at each follow-up

| | *n* | Pooled sample Mean (±SD)/Median (IQR)/n (%) | Boys Mean (±SD)/Median (IQR)/n (%) | Girls Mean (±SD)/Median (IQR)/n (%) | p for sex difference |
|---|---|---|---|---|---|
| **Pregnancy and birth measures (n=14,901)** | | | | | |
| Maternal age (years at birth) | 12921 | 28 (5) | 28.1 (5) | 27.9 (4.9) | 0.009 |
| Maternal smoking status n (%) yes | 11052 | 2157 (19.5%) | 1144 (20.2%) | 1013 (18.8%) | 0.08 |
| Gestation length (weeks) | 12921 | 39.8 (1.3) | 39.7 (1.3) | 39.8 (1.3) | <0.001 |
| Number of events | 12285 | 3.6 (2.3) | 3.6 (2.3) | 3.6 (2.3) | 0.4 |
| Perceived impact score | 12285 | 8.5 (7) | 8.4 (7) | 8.6 (7.1) | 0.2 |
| Birthweight (g) | 12766 | 3469 (478) | 3530 (490) | 3404 (457) | <0.001 |
| Breastfed (% yes) | 10359 | 6185 (59.7%) | 3132 (59%) | 3053 (60.5%) | 0.1 |
| **Offspring 7-year follow-up (n=7431)** | | | | | |
| Systolic BP | 7065 | 98.8 (9.2) | 98.7 (9.1) | 98.9 (9.3) | 0.4 |
| Diastolic BP | 7063 | 56.5 (6.7) | 56.1 (6.7) | 56.9 (6.6) | <0.001 |
| HR | 7062 | 83.3 (10.7) | 82 (10.5) | 84.6 (10.8) | <0.001 |
| **Offspring 17-year follow-up (n=5,215)** | | | | | |
| Systolic BP | 4104 | 116.4 (9.9) | 122 (9.2) | 112 (8.1) | <0.001 |
| Diastolic BP | 4104 | 64.2 (6) | 63.3 (6) | 64.9 (5.9) | <0.001 |
| HR | 4104 | 65.2 (9.7) | 62.5 (9.2) | 67.2 (9.6) | <0.001 |
| cIMT | 4102 | 0.48 (0.05) | 0.48 (0.05) | 0.47 (0.04) | <0.001 |
| PWV | 3423 | 5.8 (0.7) | 6 (0.7) | 5.6 (0.6) | <0.001 |

*SES=socioeconomic status, BP=Blood pressure (mmHg), cIMT=carotid intermedia thickness, HR= heart rate (beat per minute), PWV= Pulse wave velocity*

*Maternal smoking is yes/no smoked cigarettes regularly in the last 2 months of pregnancy*

*Breastfeeding is yes/no 1+ months of breastfeeding*

For peer review only

**Table 2:** Associations between maternal adversity and offspring CV measures

| | | *Pooled* | *Boys* | *Girls* |
|---|---|---|---|---|
| **Outcome** | **Exposure** | **Fold change (95% CI)** | **Fold change (95% CI)** | **Fold change (95% CI)** |
| Resting heart rate (bpm) | *Perceived impact score* | 0.999 (0.998-1.001) | 1.000 (0.997-1.002) | 0.999 (0.997-1.001) |
| | *Adversity number* | 0.997 (0.992-1.003) | 0.998 (0.990-1.005) | 0.996 (0.989-1.004) |
| Systolic blood pressure (mmHg) | *Perceived impact score* | 1.000 (0.999-1.001) | 1.000 (0.998-1.002) | 1.000 (0.998-1.001) |
| | *Adversity number* | 0.998 (0.994-1.001) | 0.998 (0.993-1.003) | 0.998 (0.993-1.002) |
| Diastolic blood pressure (mmHg) | *Perceived impact score* | 1.000 (0.999-1.002) | 0.999 (0.997-1.002) | 1.001 (0.999-1.003) |
| | *Adversity number* | 0.999 (0.994-1.003) | 0.996 (0.989-1.002) | 1.001 (0.996-1.007) |
| Pulse-wave velocity | *Perceived impact score* | 0.999 (0.997-1.001) | 0.999 (0.997-1.001) | 1.001 (0.998-1.004) |
| | *Adversity number* | 0.998 (0.991-1.005) | 0.9928 (0.982-1.004) | 1.001 (0.992-1.010) |
| Carotid Intima Media Thickness | *Perceived impact score* | 1.000 (0.998-1.002) | 1.001 (0.998-1.004) | 1.000 (0.997-1.002) |
| | *Adversity number* | 1.001 (0.996-1.007) | 1.001 (0.993-1.010) | 1.001 (0.994-1.008) |

Models adjusted for child age, alcohol use in pregnancy; tobacco use in pregnancy; ethnic group; parity; age at delivery; and maternal education.

NB: Pulse wave velocity and Carotid Intima Media Thickness were only measured at one time point (17 years of age)**.** Fold changes corresponds to a 4 unit change in adversity measures.

For peer review only

**Table 3: Specific maternal adversities and longitudinal offspring CV measures**

| ***Adversity event*** | *Heart rate* | | | *Systolic blood pressure* | | | *Diastolic blood pressure* | | |
|---|---|---|---|---|---|---|---|---|---|
| | **Pooled** | **Boys** | **Girl** | **Pooled** | **Boys** | **Girls** | **Pooled** | **Boys** | **Girls** |
| | **Fold change (95% CI)** | **Fold change (95% CI)** | **Fold change (95% CI)** | **Fold change (95% CI)** | **Fold change (95% CI)** | **Fold change (95% CI)** | **Fold change (95% CI)** | **Fold change (95% CI)** | **Fold change (95% CI)** |
| Partner died | 0.93 (0.80-1.09) | 0.93 (0.76-1.15) | 0.94 (0.74-1.18) | 0.98 (0.90-1.07) | 0.93 (0.83-1.05) | 1.04 (0.90-1.19) | 0.99 (0.89-1.11) | 0.95 (0.82-1.10) | 1.06 (0.90-1.26) |
| Child died | 1.06 (0.85-1.33) | 1.06 (0.71-1.57) | 1.06 (0.81-1.40) | 1.10 (0.94-1.27) | 1.08 (0.82-1.41) | 1.11 (0.94-1.33) | 1.15 (0.96-1.38) | 1.18 (0.84-1.67) | 1.14 (0.92-1.41) |
| Friend or relative died | 1.00 (0.99-1.02) | 1.01 (0.99-1.04) | 0.99 (0.97-1.02) | 0.99 (0.98-1.00) | 1.00 (0.98-1.01) | 0.99 (0.98-1.00) | 1.00 (0.99-1.01) | 1.00 (0.98-1.01) | 1.00 (0.99-1.02) |
| Child was ill | 0.99 (0.98-1.01) | 0.99 (0.98-1.02) | 0.99 (0.97-1.01) | 1.00 (0.99-1.01) | 1.00 (0.99-1.02) | 0.99 (0.98-1.00) | 1.00 (0.98-1.01) | 1.00 (0.98-1.02) | 0.99 (0.98-1.01) |
| Partner was ill | 1.00 (0.98-1.02) | 1.00 (0.97-1.03) | 1.00 (0.97-1.03) | 0.99 (0.98-1.00) | 0.98 (0.97-1.00) | 1.00 (0.98-1.02) | 1.00 (0.98-1.01) | 0.99 (0.96-1.01) | 1.01 (0.99-1.03) |
| Friend or relative was ill | 1.00 (0.98-1.01) | 1.01 (0.99-1.03) | 0.99 (0.97-1.01) | 1.00 (0.99-1.01) | 1.00 (0.99-1.01) | 1.00 (0.99-1.01) | 1.01 (0.99-1.02) | 1.01 (0.99-1.02) | 1.00 (0.99-1.02) |
| Admitted to hospital | 0.99 (0.98-1.01) | 1.01 (0.99-1.03) | 0.97 (0.96-0.99)# | 1.00 (0.99-1.00) | 1.00 (0.99-1.02) | 0.99 (0.98-1.00)# | 1.00 (0.99-1.01) | 1.00 (0.99-1.02) | 0.99 (0.97-1.01) |
| In trouble with the law | 1.02 (0.93-1.11) | 1.08 (0.95-1.24) | 0.97 (0.86-1.09) | 1.00 (0.96-1.05) | 0.91 (0.84-0.99)# | 1.06 (1.00-1.12) | 1.01 (0.95-1.07) | 0.97 (0.88-1.07) | 1.04 (0.97-1.11) |
| Divorced | 1.02 (0.95-1.10) | 1.05 (0.96-1.15) | 0.99 (0.89-1.10) | 1.04 (1.00-1.08) | 1.03 (0.97-1.09) | 1.06 (1.00-1.12) | 1.05 (0.99-1.10) | 1.04 (0.97-1.11) | 1.05 (0.98-1.13) |
| Partner rejected pregnancy | 1.00 (0.97-1.03) | 1.01 (0.97-1.05) | 1.00 (0.96-1.03) | 0.99 (0.98-1.01) | 0.98 (0.96-1.00) | 1.01 (0.99-1.03) | 1.01 (0.99-1.03) | 0.99 (0.97-1.02) | 1.02 (1.00-1.05) |
| Very ill | 1.00 (0.98-1.02) | 1.02 (0.99-1.04) | 1.00 (0.98-1.03) | 1.01 (1.00-1.02) | 1.01 (0.99-1.03) | 1.01 (0.99-1.03) | 1.02 (1.01-1.04)* | 1.03 (1.01-1.05)* | 1.01 (0.99-1.03) |
| Partner lost job | 1.00 (0.98-1.01) | 1.00 (0.97-1.03) | 0.99 (0.97-1.02) | 1.00 (0.99-1.01) | 1.01 (0.99-1.02) | 1.00 (0.98-1.02) | 1.01 (1.00-1.03) | 1.01 (0.99-1.04) | 1.01 (0.99-1.03) |
| Partner had problems at work | 0.99 (0.98-1.01) | 0.99 (0.97-1.01) | 1.00 (0.98-1.01) | 1.00 (0.99-1.01) | 1.00 (0.99-1.01) | 1.00 (0.99-1.01) | 1.00 (0.99-1.01) | 0.99 (0.97-1.00) | 1.00 (0.99-1.01) |
| Problems at work | 1.00 (0.98-1.01) | 0.99 (0.98-1.02) | 1.00 (0.97-1.02) | 1.01 (1.00-1.02) | 1.01 (0.99-1.03) | 1.01 (0.99-1.02) | 1.01 (1.00-1.02) | 1.01 (0.99-1.03) | 1.02 (1.00-1.03) |
| Lost job | 0.99 (0.96-1.02) | 0.97 (0.93-1.02) | 1.00 (0.96-1.04) | 1.00 (0.98-1.02) | 1.01 (0087-1.04) | 1.00 (0.98-1.03) | 1.01 (0.99-1.03) | 1.01 (0.98-1.05) | 1.01 (0.98-1.04) |
| Partner went away | 1.00 (0.98-1.02) | 1.00 (0.97-1.02) | 1.00 (0.98-1.02) | 1.00 (0.99-1.01) | 0.99 (0.98-1.01) | 1.00 (0.99-1.02) | 1.00 (0.98-1.01) | 0.99 (0.97-1.01) | 1.00 (0.99-1.02) |
| Partner in trouble with law | 0.98 (0.95-1.02) | 1.00 (0.95-1.06) | 0.97 (0.92-1.02) | 1.00 (0.98-1.03) | 0.99 (0.95-1.03) | 1.02 (0.99-1.06) | 0.98 (0.95-1.01) | 0.95 (0.91-1.00)# | 1.00 (0.96-1.04) |
| Separated | 1.01 (0.99-1.03) | 1.01 (0.98-1.04) | 1.01 (0.98-1.04) | 1.01 (0.99-1.02) | 0.99 (0.97-1.01) | 1.03 (1.01-1.04)* | 1.00 (0.98-1.02) | 0.99 (0.96-1.02) | 1.01 (0.99-1.04) |
| Income reduced | 1.00 (0.99-1.01) | 1.01 (0.99-1.02) | 0.99 (0.98-1.00) | 1.00 (1.00-1.01) | 1.01 (1.00-1.02)# | 1.00 (0.99-1.01) | 1.01 (1.00-1.02) | 1.01 (1.00-1.03) | 1.01 (0.99-1.02) |
| Argued with partner | 0.99 (0.98-1.00) | 1.00 (0.99-1.02) | 0.98 (0.97-1.00 # | 1.00 (0.99-1.00) | 1.00 (0.99-1.01) | 0.99 (0.98-1.00) | 1.00 (0.99-1.01) | 1.00 (0.99-1.01) | 0.99 (0.98-1.00) |
| Argued with family or friends | 1.00 (0.98-1.01) | 1.00 (0.98-1.02) | 1.00 (0.98-1.02) | 1.00 (0.99-1.01) | 1.00 (0.99-1.02) | 0.99 (0.98-1.00) | 1.00 (0.99-1.01) | 1.00 (0.98-1.01) | 1.00 (0.99-1.02) |
| Moved house | 1.02 (1.00-1.03) | 1.01 (0.99-1.04) | 1.02 (1.00-1.04)# | 1.00 (0.99-1.01) | 1.00 (0.99-1.02) | 1.00 (0.99-1.01) | 0.99 (0.98-1.01) | 0.99 (0.97-1.01) | 1.00 (0.98-1.01) |
| Partner hurt mother | 0.99 (0.95-1.03) | 0.99 (0.94-1.05) | 0.99 (0.94-1.04) | 0.99 (0.97-1.02) | 0.98 (0.95-1.02) | 1.01 (0.98-1.04) | 0.97 (0.94-1.00)# | 0.95 (0.90-0.99)# | 0.98 (0.94-1.02) |

For peer review only



For peer review only - http://bmjopen.bmj.com/site/about/guidelines.xhtml

| | | | | | | | | | |
|---|---|---|---|---|---|---|---|---|---|
| Became homeless | 1.00 (0.97-1.04) | 1.00 (0.95-1.06) | 1.00 (0.95-1.05) | 1.00 (0.98-1.03) | 0.99 (0.96-1.03) | 1.01 (0.98-1.05) | 0.97 (0.94-1.00)$^{\#}$ | 0.94 (0.90-0.98)$^{*}$ | 1.00 (0.96-1.04) |
| Major financial problems | 1.01 (1.00-1.02) | 1.01 (0.99-1.03) | 1.01 (0.99-1.02) | 1.01 (1.00-1.02) | 1.01 (1.00-1.02) | 1.01 (1.00-1.02) | 1.01 (1.00-1.02) | 1.01 (0.99-1.02) | 1.01 (1.00-1.03)$^{\#}$ |
| Got married | 1.00 (0.97-1.03) | 0.98 (0.94-1.03) | 1.02 (0.97-1.07) | 1.00 (0.97-1.01) | 1.00 (0.97-1.02) | 0.99 (0.96-1.02) | 1.00 (0.98-1.03) | 1.00 (0.96-1.03) | 1.01 (0.97-1.05) |
| Partner hurt child | 0.94 (0.82-1.08) | 0.73 (0.59-0.91)$^{\wedge}$ | 1.11 (0.94-1.32) | 1.03 (0.94-1.11) | 0.93 (0.81-1.07) | 1.06 (0.96-1.18) | 0.95 (0.85-1.05) | 0.87 (0.73-1.04) | 1.00 (0.88-1.14) |
| Attempted suicide | 1.02 (1.05-1.39)$^{*}$ | 1.26 (1.02 -1.56)$^{\#}$ | 1.16 (0.95-1.40) | 0.97 (0.88-1.06) | 1.01 (0.88-1.16) | 0.96 (0.85-1.08) | 1.07 (0.96-1.21) | 1.09 (0.92-1.31) | 1.06 (0.91-1.23) |
| Convicted of an offence | 1.09 (0.97-1.23) | 1.14 (0.93-1.39) | 1.07 (0.93-1.23) | 0.99 (0.92-1.07) | 0.86 (0.75-0.98)$^{\#}$ | 1.07 (0.97-1.17) | 1.02 (0.93-1.12) | 0.98 (0.83-1.15) | 1.04 (0.94-1.17) |
| Bled & thought might miscarry | 0.99 (0.97-1.00) | 0.99 (0.97-1.01) | 0.99 (0.97-1.01) | 1.00 (1.00-1.01) | 1.00 (0.99-1.02) | 1.00 (0.99-1.02) | 1.00 (0.99-1.01) | 1.00 (0.98-1.01) | 1.00 (0.99-1.02) |
| Started new job | 1.00 (0.97-1.04) | 0.99 (0.94-1.04) | 1.01 (0.97-1.06) | 0.99 (0.97-1.02) | 0.99 (0.95-1.02) | 0.99 (0.96-1.02) | 1.00 (0.99-1.01) | 0.99 (0.97-1.00) | 1.00 (0.99-1.01) |
| Test to see if baby abnormal | 1.00 (0.98-1.01) | 0.99 (0.97-1.01) | 1.00 (0.98-1.02) | 1.00 (0.99-1.01) | 0.99 (0.98-1.00) | 1.00 (0.99-1.01) | 1.00 (0.99-1.01) | 0.99 (0.97-1.00) | 1.00 (0.99-1.02) |
| Tests show baby possibly abnormal | 1.00 (0.97-1.02) | 0.99 (0.96-1.02) | 1.01 (0.98-1.04) | 1.00 (0.98-1.01) | 0.99 (0.96-1.01) | 1.01 (0.99-1.03) | 1.00 (0.98-1.02) | 0.99 (0.96-1.01) | 1.01 (0.98-1.04) |
| Told having twins | 1.05 (1.00-1.11) | 1.03 (0.95-1.10) | 1.09 (1.00-1.19)$^{\#}$ | 0.99 (0.96-1.03) | 0.99 (0.95-1.04) | 0.99 (0.94-1.04) | 0.98 (0.94-1.02) | 0.97 (0.92-1.03) | 0.99 (0.92-1.06) |
| Possible harm to baby | 0.99 (0.97-1.01) | 0.98 (0.95-1.00) | 1.00 (0.97-1.02) | 1.00 (0.99-1.02) | 1.00 (0.98-1.01) | 1.01 (0.99-1.03) | 1.00 (0.99-1.02) | 0.99 (0.97-1.02) | 1.02 (0.99-1.04) |
| Tried to have abortion | 1.04 (0.98-1.10) | 1.04 (0.96-1.14) | 1.03 (0.95-1.12) | 1.01 (0.98-1.06) | 0.99 (0.94-1.05) | 1.04 (0.98-1.09) | 1.02 (0.98-1.08) | 1.03 (0.96-1.11) | 1.03 (0.96-1.10) |
| Took an exam | 1.01 (0.99-1.04) | 1.04 (1.00-1.09) | 0.99 (0.95-1.03)$^{\#}$ | 0.99 (0.97-1.01) | 1.01 (0.98-1.04) | 0.97 (0.94-0.99)$^{*}$ | 0.99 (0.97-1.01) | 1.01 (0.97-1.04) | 0.98 (0.94-1.01) |
| Partner emotionally cruel to mother | 0.99 (0.97-1.01) | 0.98 (0.96-1.00) | 1.00 (0.97-1.02) | 1.00 (0.99-1.02) | 1.01 (0.99-1.02) | 1.00 (0.98-1.02) | 1.01 (0.99-1.02) | 1.00 (0.91-1.02) | 1.01 (0.99-1.03) |
| Partner emotionally cruel to child | 0.99 (0.95-1.04) | 1.01 (0.93-1.09) | 0.98 (0.91-1.05) | 0.99 (0.96-1.02) | 0.98 (0.93-1.03) | 0.99 (0.96-1.04) | 0.97 (0.94-1.01) | 1.01 (0.94-1.08) | 0.96 (0.91-1.00) |
| House or car burgled | 1.00 (0.97-1.01) | 0.98 (0.95-1.01) | 1.00 (0.97-1.03) | 0.99 (0.97-1.00) | 1.00 (0.97-1.02) | 0.99 (0.97-1.01) | 0.99 (0.98-1.01) | 0.98 (0.96-1.01) | 1.00 (0.97-1.02) |
| Had an accident | 1.01 (0.92-1.05) | 1.00 (0.95-1.04) | 1.03 (1.00-1.08) | 0.99 (0.97-1.01) | 0.98 (0.96-1.01) | 0.99 (0.96-1.02) | 1.00 (0.97-1.02) | 0.99 (0.96-1.03) | 1.00 (0.96-1.03) |

# denotes $p \leq 0.05$

* denotes $p \leq 0.01$

^ denotes $p \leq 0.001$

**NB:** Fold changes represent a 4 unit change.

For peer review only



For peer review only - http://bmjopen.bmj.com/site/about/guidelines.xhtml

**Figure 1:** The relationship between CpG probe cg20222926 and offspring diastolic blood pressure

For peer review only



For peer review only - http://bmjopen.bmj.com/site/about/guidelines.xhtml

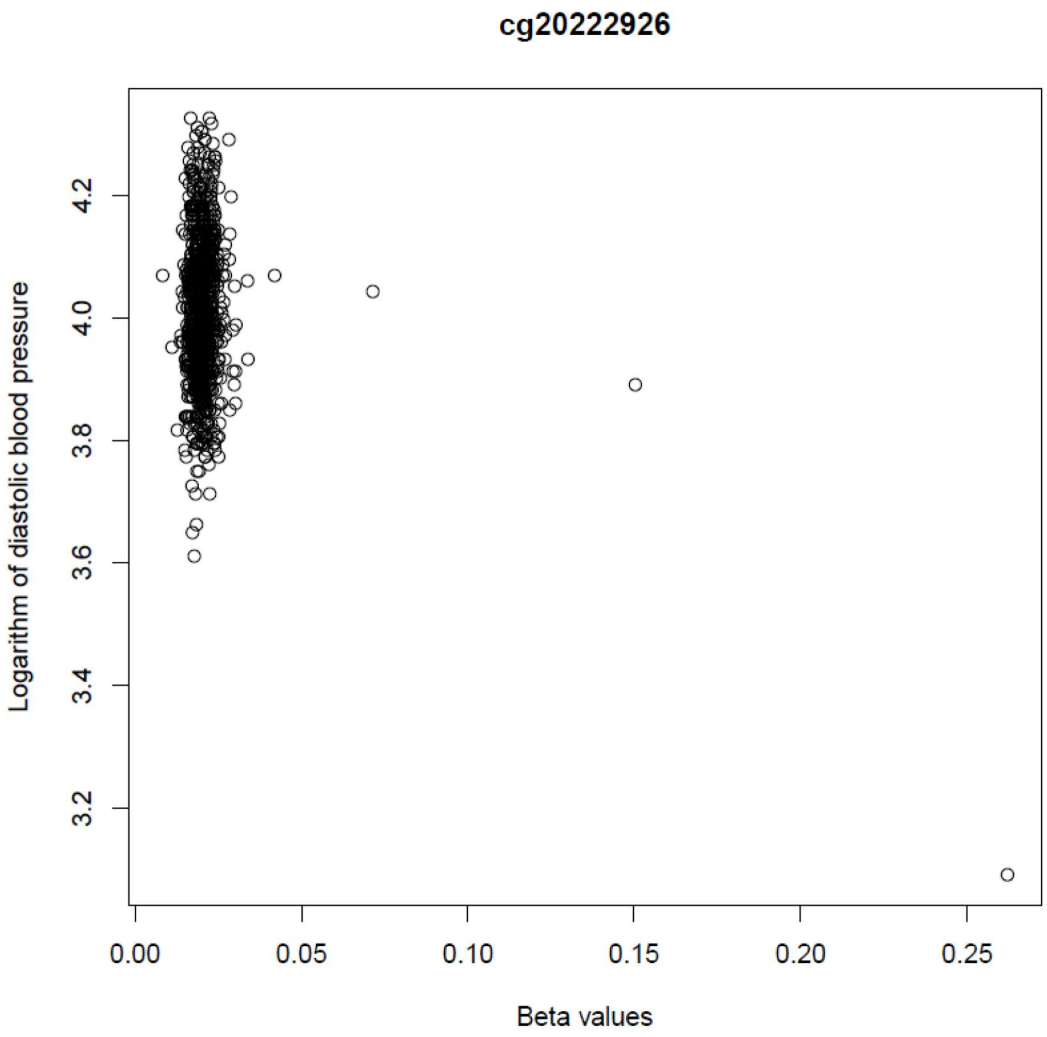


**Figure 1:** The relationship between CpG probe cg20222926 and offspring diastolic blood pressure

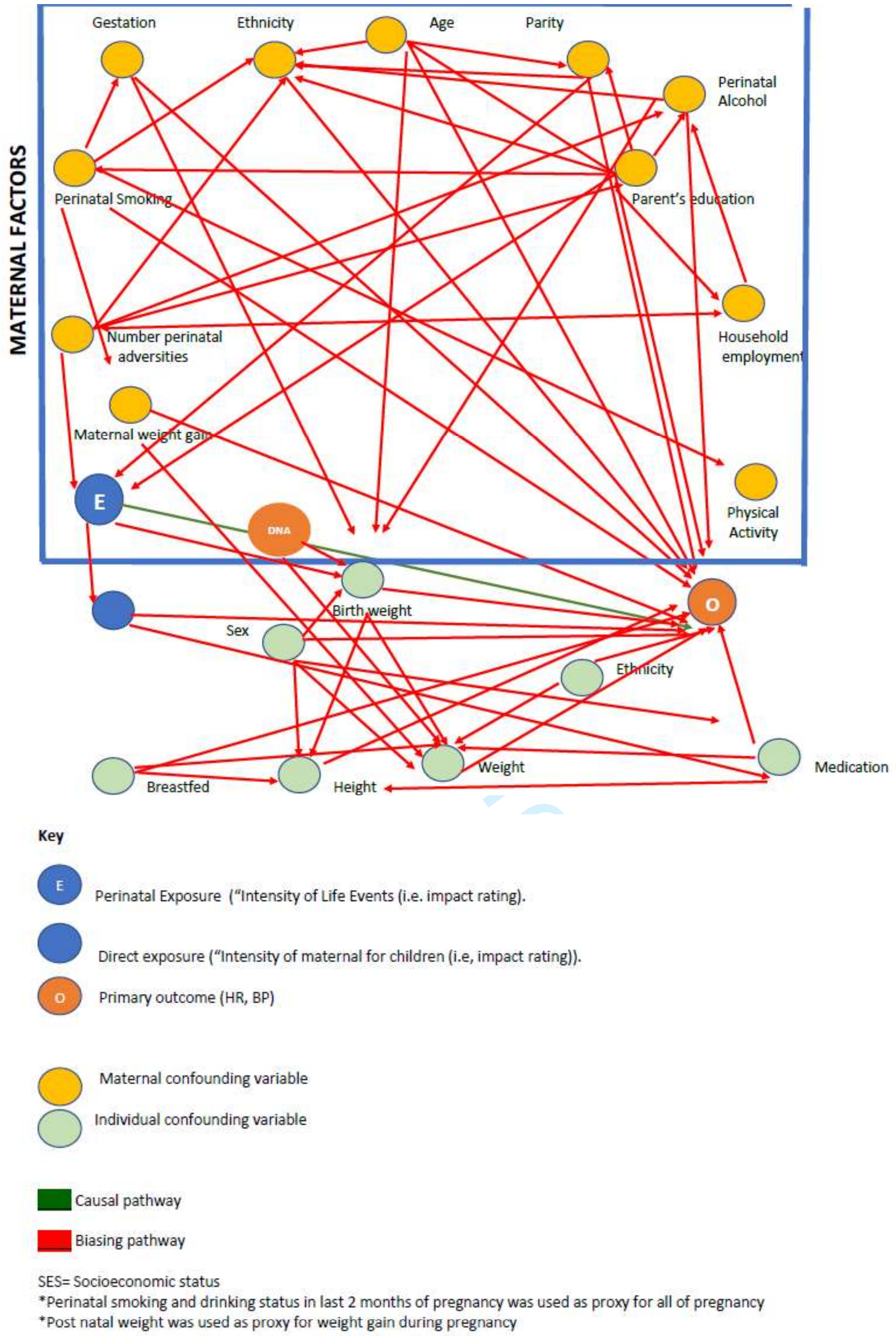


**Supplementary Figure S1:** Directed Acyclic Graph at 7 years of age

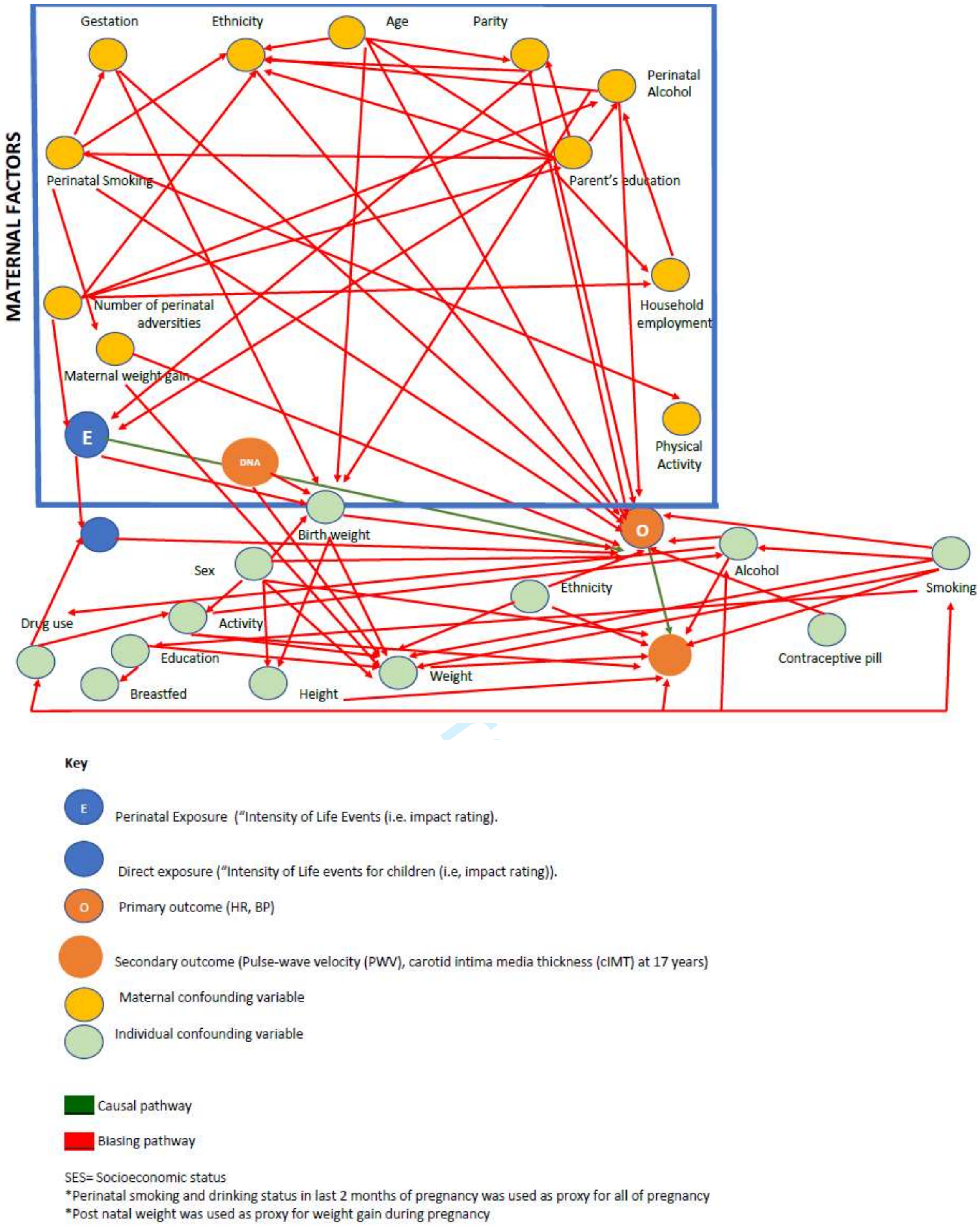


**Supplementary Figure S2:** Directed Acyclic Graph at seventeen years of age

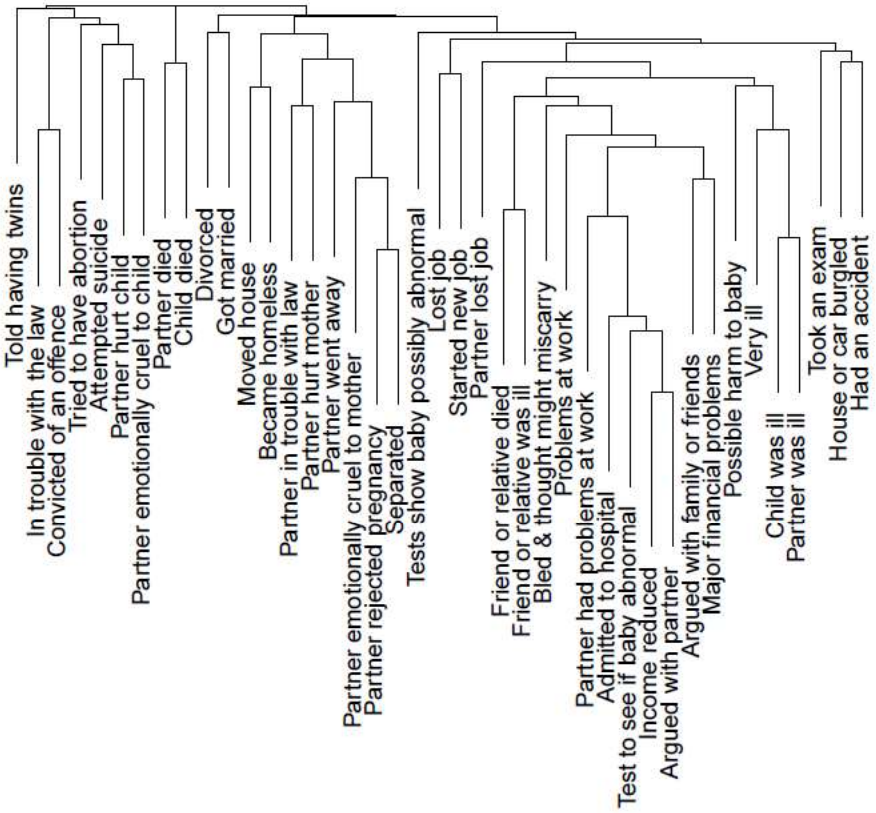


**Supplementary Figure S3:** Dendrogram of specific life events clusters

STROBE Statement—checklist of items that should be included in reports of observational studies

| | Item No | Recommendation | Page No |
|---|---|---|---|
| **Title and abstract** | 1 | (*a*) Indicate the study's design with a commonly used term in the title or the abstract | 2 |
| | | (*b*) Provide in the abstract an informative and balanced summary of what was done and what was found | 2 |
| **Introduction** | | | |
| Background/rationale | 2 | Explain the scientific background and rationale for the investigation being reported | 4-5 |
| Objectives | 3 | State specific objectives, including any prespecified hypotheses | 5 |
| **Methods** | | | |
| Study design | 4 | Present key elements of study design early in the paper | 6-9 |
| Setting | 5 | Describe the setting, locations, and relevant dates, including periods of recruitment, exposure, follow-up, and data collection | 6-8 |
| Participants | 6 | (*a*) *Cohort study*—Give the eligibility criteria, and the sources and methods of selection of participants. Describe methods of follow-up *Case-control study*—Give the eligibility criteria, and the sources and methods of case ascertainment and control selection. Give the rationale for the choice of cases and controls *Cross-sectional study*—Give the eligibility criteria, and the sources and methods of selection of participants | 6 |
| | | (*b*) *Cohort study*—For matched studies, give matching criteria and number of exposed and unexposed *Case-control study*—For matched studies, give matching criteria and the number of controls per case | N/A |
| Variables | 7 | Clearly define all outcomes, exposures, predictors, potential confounders, and effect modifiers. Give diagnostic criteria, if applicable | 6-8 |
| Data sources/ measurement | 8* | For each variable of interest, give sources of data and details of methods of assessment (measurement). Describe comparability of assessment methods if there is more than one group | 6-8 |
| Bias | 9 | Describe any efforts to address potential sources of bias | 15 |
| Study size | 10 | Explain how the study size was arrived at | 6 |
| Quantitative variables | 11 | Explain how quantitative variables were handled in the analyses. If applicable, describe which groupings were chosen and why | 8 |
| Statistical methods | 12 | (*a*) Describe all statistical methods, including those used to control for confounding | 8 |
| | | (*b*) Describe any methods used to examine subgroups and interactions | 8 |
| | | (*c*) Explain how missing data were addressed | 6 |
| | | (*d*) *Cohort study*—If applicable, explain how loss to follow-up was addressed *Case-control study*—If applicable, explain how matching of cases and controls was addressed *Cross-sectional study*—If applicable, describe analytical methods taking account of sampling strategy | 6 |
| | | (*e*) Describe any sensitivity analyses | N/A |

Continued on next page



For peer review only - http://bmjopen.bmj.com/site/about/guidelines.xhtml

For peer review only

| | | | |
|---|---|---|---|
| **Results** | | | |
| Participants | 13* | (a) Report numbers of individuals at each stage of study—eg numbers potentially eligible, examined for eligibility, confirmed eligible, included in the study, completing follow-up, and analysed | 6 |
| | | (b) Give reasons for non-participation at each stage | 6 |
| | | (c) Consider use of a flow diagram | - |
| Descriptive data | 14* | (a) Give characteristics of study participants (eg demographic, clinical, social) and information on exposures and potential confounders | 19 |
| | | (b) Indicate number of participants with missing data for each variable of interest | Tab 1 |
| | | (c) *Cohort study*—Summarise follow-up time (eg, average and total amount) | |
| Outcome data | 15* | *Cohort study*—Report numbers of outcome events or summary measures over time | Tab 1 |
| | | *Case-control study*—Report numbers in each exposure category, or summary measures of exposure | - |
| | | *Cross-sectional study*—Report numbers of outcome events or summary measures | - |
| Main results | 16 | (*a*) Give unadjusted estimates and, if applicable, confounder-adjusted estimates and their precision (eg, 95% confidence interval). Make clear which confounders were adjusted for and why they were included | 8 |
| | | (*b*) Report category boundaries when continuous variables were categorized | - |
| | | (*c*) If relevant, consider translating estimates of relative risk into absolute risk for a meaningful time period | - |
| Other analyses | 17 | Report other analyses done—eg analyses of subgroups and interactions, and sensitivity analyses | 8 |
| **Discussion** | | | |
| Key results | 18 | Summarise key results with reference to study objectives | 13 |
| Limitations | 19 | Discuss limitations of the study, taking into account sources of potential bias or imprecision. Discuss both direction and magnitude of any potential bias | 15 |
| Interpretation | 20 | Give a cautious overall interpretation of results considering objectives, limitations, multiplicity of analyses, results from similar studies, and other relevant evidence | 15 |
| Generalisability | 21 | Discuss the generalisability (external validity) of the study results | 15 |
| **Other information** | | | |
| Funding | 22 | Give the source of funding and the role of the funders for the present study and, if applicable, for the original study on which the present article is based | 16 |

*Give information separately for cases and controls in case-control studies and, if applicable, for exposed and unexposed groups in cohort and cross-sectional studies.

**Note:** An Explanation and Elaboration article discusses each checklist item and gives methodological background and published examples of transparent reporting. The STROBE checklist is best used in conjunction with this article (freely available on the Web sites of PLoS Medicine at http://www.plosmedicine.org/, Annals of Internal Medicine at http://www.annals.org/, and Epidemiology at http://www.epidem.com/). Information on the STROBE Initiative is available at www.strobe-statement.org.



For peer review only - http://bmjopen.bmj.com/site/about/guidelines.xhtml

BMJ Open

# The association between maternal adversity, DNA methylation, and cardiovascular health of offspring: a longitudinal analysis of the ALSPAC cohort study

| | |
|---|---|
| Journal: | *BMJ Open* |
| Manuscript ID | bmjopen-2021-053652.R1 |
| Article Type: | Original research |
| Date Submitted by the Author: | 26-Jan-2022 |
| Complete List of Authors: | Hyde, Natalie; Deakin University, School of Medicine<br>Dowty, James; The University of Melbourne, Centre for Epidemiology and Biostatistics<br>Scovelle, Anna; University of Melbourne VCCC,<br>Armstrong, Gregory; University of Melbourne, Centre for Mental Health, Melbourne School of Population and Global Health<br>Sutherland, Georgina; The University of Melbourne, Centre for Mental Health<br>Olive, Lisa; Deakin, School of Pyschology<br>Lycett, kate; Murdoch Childrens Research Institute, Community Child Health; Deakin, School of Pyschology<br>O'Neil, Adrienne ; Deakin, School of Medicine |
| <b>Primary Subject Heading</b>: | Cardiovascular medicine |
| Secondary Subject Heading: | Epidemiology |
| Keywords: | CARDIOLOGY, PSYCHIATRY, PERINATOLOGY |
| | |

SCHOLARONE™ Manuscripts

*I, the Submitting Author has the right to grant and does grant on behalf of all authors of the Work (as defined in the below author licence), an exclusive licence and/or a non-exclusive licence for contributions from authors who are: i) UK Crown employees; ii) where BMJ has agreed a CC-BY licence shall apply, and/or iii) in accordance with the terms applicable for US Federal Government officers or employees acting as part of their official duties; on a worldwide, perpetual, irrevocable, royalty-free basis to BMJ Publishing Group Ltd ("BMJ") its licensees and where the relevant Journal is co-owned by BMJ to the co-owners of the Journal, to publish the Work in this journal and any other BMJ products and to exploit all rights, as set out in our licence.*

*The Submitting Author accepts and understands that any supply made under these terms is made by BMJ to the Submitting Author unless you are acting as an employee on behalf of your employer or a postgraduate student of an affiliated institution which is paying any applicable article publishing charge ("APC") for Open Access articles. Where the Submitting Author wishes to make the Work available on an Open Access basis (and intends to pay the relevant APC), the terms of reuse of such Open Access shall be governed by a Creative Commons licence – details of these licences and which Creative Commons licence will apply to this Work are set out in our licence referred to above.*

*Other than as permitted in any relevant BMJ Author's Self Archiving Policies, I confirm this Work has not been accepted for publication elsewhere, is not being considered for publication elsewhere and does not duplicate material already published. I confirm all authors consent to publication of this Work and authorise the granting of this licence.*

For peer review only

# The association between maternal adversity, DNA methylation, and cardiovascular health of offspring: a longitudinal analysis of the ALSPAC cohort study

Natalie K Hyde[1*], James G Dowty[2], Anna Scovelle[3], Gregory Armstrong[4], Georgina Sutherland[5], Lisa Olive[6], Kate Lycett[6-8], Adrienne O'Neil[1]

1. Deakin University, IMPACT, the Institute for Mental and Physical Health and Clinical Translation, Barwon Health, School of Medicine, Geelong, Victoria, Australia.
2. Centre for Epidemiology and Biostatistics, The University of Melbourne, Australia
3. Gender & Health Unit, Centre for Health Equity, The University of Melbourne, Australia
4. Nossal Institute of Global Health, The University of Melbourne, Australia
5. Centre for Mental Health, The University of Melbourne, Australia
6. School of Pyschology, Deakin University, Australia
7. Population Health, Murdoch Children's Research Institute, Australia
8. Department of Pediatrics, The University of Melbourne, Australia

**[*]Corresponding author**

Natalie Hyde

Population Health, IMPACT Institute, School of Medicine, Deakin University (Barwon Health), PO Box 281

Geelong, Victoria, Australia, 3220

Email: natalie.hyde@deakin.edu.au

**Word count:**



For peer review only - http://bmjopen.bmj.com/site/about/guidelines.xhtml

Manuscript: 3,499

**Reference count: 26**

**Keywords:** adversity, ALSPAC, CV disease, epigenetic, maternal, stress

**Abstract**

**Objectives:** Maternal adversity during pregnancy has been shown to be associated with some health outcomes in the offspring. This study investigated the association of maternal adversity during pregnancy and DNA methylation with offspring cardiovascular (CV) health.

**Design:** Longitudinal observational cohort study

**Setting:** All pregnant residents in county Avon (~0.9 million), United Kingdom, were eligible to participate if their estimated delivery date was between 1 April 1991 and 31 December 1992.

**Participants:** Mother–offspring pairs enrolled in the Avon Longitudinal Study of Parents and Children (ALSPAC) cohort at seven (n=7431) and 17 years of age (n=3143).

**Primary and secondary outcome measures:** Offspring CV health primary measures were heart rate (HR), blood pressure (BP), and secondary measures were pulse-wave velocity and carotid intima media thickness.

**Results:** Overall, there was no association between maternal adversity scores (number or perceived impact) and primary CV measures (Perceived impact; HR: 0.999-fold change 95% CI 0.998,1.001; systolic BP [SBP]: 1.000-fold change 95% CI 0.999,1.001; diastolic BP: 1.000-fold change CI 0.999,1.002). Some small offspring sex effects were observed and there was also a small association between methylation of cg20111643 (TOM1L1) in cord blood and offspring SBP (1.013-fold change 95% CI: 1.008, 1.017 per standard deviation).

**Conclusions:** We found little evidence to support the overall association of maternal adversity during pregnancy and DNA methylation with offspring CV measures. Offspring sex- and age-specific associations require further investigation.

For peer review only

**Strengths and limitations of this study**

- A strength of this study is longitudinal collection of phenotypic data in both women and their child; detailed cardiovascular measures in the offspring have been collected at multiple timepoints.
- A limitation is attrition bias, with those of a higher socioeconomic status being more likely to remain in the study over time.
- In addition it is possible that some life stressors during pregnancy may have not have been captured given the list of potential stressors was not exhaustive.
- It is plausible that any effects during pregnancy may have been diluted by the inclusion of data about maternal stressors that was collected in the early postpartum phase (8 weeks post-partum).

For peer review only

## Introduction

Seminal work conducted by Barker et al. in the early twentieth century noted geographical differences in infant mortality rates, whereby regions of England with the highest infant mortality rates also had the highest rate of coronary heart disease (CHD) mortality[1 2]. From this, it was concluded "*…adverse environmental influences in utero and during infancy, associated with poor living standards, directly increased susceptibility to the disease (CHD).*" Further extending this work, the Developmental Origins of Health and Disease (DOHaD) hypothesis[3] proposes that the risk of chronic diseasesoriginate not only from an individual's genome but also by its interactions with biological insults *in utero* and early life.

To date, much of the work in this area has focussed on the impact of maternal nutrition during pregnancy, with comparatively fewer data on social adversity and trauma. However, there are some data to suggest that the ways in which women experience social adversity during pregnancy may induce similar changes to disease trajectory in the offspring as maternal malnourishment[4].

The time *in utero* represents a critical period of development, which may be particularly vulnerable to maternal stress. During this time, it is plausible the fetus is directly susceptible to the biological effects of maternal stress owing to its reliance on the maternal blood supply via the placenta. Epigenetics are mitotically heritable changes to gene expression that do not involve changes to the underlying genetic sequence. These changes in gene expression may provide some clues about the mechanisms through which maternal adversity embeds itself into an individual and her offspring. A recent review of maternal prenatal stress and infant DNA



For peer review only - http://bmjopen.bmj.com/site/about/guidelines.xhtml

methylation identified several candidate genes implicated in the maternal central stress response that may be critical in driving phenotype changes for offspring[4]. However, recent evidence also suggests that perhaps the placenta may buffer the effects of the maternal stress response[5].

The extent of cumulative damage to biological systems that occurs with increasing number, duration or severity of exposures, particularly with age, is likely to be a critical consideration in understanding associations between maternal adversity and the cardiovascular (CV) health of a child. This includes distinguishing the response (e.g., perceived stress) from the stimulus or stressor (e.g., the adversity) itself. It is also notable that there appears to be a sexually dimorphic response with regards to several developmental exposures and cardiovascular conditions[6]. These issues, along with other key gaps in the evidence base that exist in psycho-cardiology have been outlined in the position paper by the American Heart Association (AHA)[7]. Specifically, these gaps relate to, (i) an absence of truly prospective studies that commenced in the pre-natal period with capacity to explicate this relationship, and (ii) a lack of studies that identify the biological mechanisms linking adversity to CVD.

This study therefore seeks to, (i) investigate the respective and cumulative impact of women's exposure(s) to adversity during the perinatal period, and cord blood DNA methylation on CV health of her offspring, (ii) establish whether associations are sex or age-dependent, and (iii) determine whether DNA methylation at birth is associated with CV outcomes. We hypothesise that greater maternal adversity will be associated with poorer CV health of offspring and DNA methylation will be associated with offspring CV measures.



For peer review only - http://bmjopen.bmj.com/site/about/guidelines.xhtml

For peer review only

## Methods

### Study design and participants

This study used longitudinal data from the Avon Longitudinal Study of Parents and Children (ALSPAC formerly "Children of the 90s" study). ALSPAC is a prospective birth cohort study conducted in the United Kingdom. The full study protocol is available elsewhere with participation rates and reason for not participation [8 9]. Briefly, all pregnant women residing in county Avon (~0.9 million) were eligible to participate if their estimated delivery date was between 1 April 1991 and 31 December 1992 inclusive. Recruitment occurred via maternity health services and mass media campaigns. After their initial expression of interest and assessment of eligibility by ALSPAC staff, women were sent the baseline questionnaire ~1 week later. The women of 14,541 pregnancies (71.8% of all pregnancies in the area at that time) were recruited antenatally during 1990–92. They completed a series of postal questionnaires throughout their pregnancy and there were several clinical assessments post-birth. CV health data were collected when the children were aged seven and 17 years.

There were 13,617 mother-offspring pairs from singleton live births who survived to ≥1 year of age; only singleton pregnancies and those women with term deliveries were included in the analyses. When the oldest children were approximately 7 years of age, an attempt was made to bolster the initial sample with eligible cases who had failed to join the study originally, resulting in an additional 913 children being enrolled. The total sample size for analyses using any data collected after the age of seven is therefore 15,454 pregnancies, resulting in 15,589 foetuses. Of these 14,901 were alive at 1 year of age. The number of children with CV measures at the subsequent 7- and 17-year time points were 7431 and 5215 (Figure 1), respectively and were included in analyses if they had complete information for the relevant analyses.

### Measures



For peer review only - http://bmjopen.bmj.com/site/about/guidelines.xhtml

*Exposure variable*. Data were those provided by women at (i) 0-18 weeks gestation, and (ii) between 19-weeks gestation and 8-weeks postpartum. Women retrospectively self-reported social adversities and rated its impact for the respective period. Adverse life events were assessed using a 41-item self-report questionnaire based on a Life Events Inventory[10], using the average score at the two timepoints. The internal reliability of the inventory, as indicated by the coefficient, is 0.68. Each item was rated in one of five categories: "Yes, affected me a lot," "Yes, affected me moderately," "Yes, affected me mildly," "Yes, but did not affect me," and "No, did not happen" and was rated from 0 to 4, with higher scores indicating greater perceived stress. Two scores were calculated as follows: (1) the number of stressful life and (2) the perceived impact of the events.

*Outcome variables*. The primary outcomes were blood pressure and heart rate at 7 and 17 years of age. Duplicate measures of resting heart rate, systolic BP (SBP) and diastolic BP (DBP) were taken using a Dinamap 9301 Vital Signs Monitor whilst participants were seated, using the average of the two readings.

Secondary outcomes were pulse-wave velocity (PWV) and carotid intima media thickness (cIMT) at 17 years of age, using the mean of three measures and the mean of three end-diastolic measurements of both the left and right side, respectively. A Vicorder device (Skidmore Medical, UK) was used to measure PWV and a Zonare Z.OneUltra system that had a a L10-5 linear transducer (Zonare Medical Systems, CA, US) was used to determine cIMT. Detailed protocols have been described elsewhere[11].

*DNA methylation data.* Embedded within the ALSPAC study is a human epigenetic resource; the Accessible Resource for Integrated Epigenomic Studies (ARIES)[12]. Of the 1018 mother–



For peer review only - http://bmjopen.bmj.com/site/about/guidelines.xhtml

offspring pairs in the ARIES project, 916 offspring had cord blood methylation data, which passed quality control [13]. Venous cord blood at birth was used to assess epigenome-wide methylation levels using the Illumina Infinium® HumanMethylation450 (HM450) BeadChip. Raw intensity signals were processed and M-values were calculated using the minfi package[14]. Probes and samples were removed if they failed quality assurance based on their detection p-values. All samples were Illumina and SWAN normalised to reduce technical bias between Type 1 and Type 2 probes.

*Confounding variables.* Directed acyclic graphs (DAGs) were constructed (Supplementary Figures S1 and S2) from which a minimal set of adjusted variables were selected using the R packages ggdag and dagitty. In the primary analyses, the final models were adjusted for child age, alcohol use in pregnancy, tobacco use in pregnancy, ethnic group, parity, age at delivery, and maternal education. All methylation analyses were additionally adjusted for white blood cell composition, using the algorithm by Houseman et al.[15].

**Statistical analyses**

Outcomes were log-transformed for anaylses. Linear mixed models[16] with random intercepts (one for each offspring) were used to analyse the association between these longitudinal outcome variables and various exposure variables (individual adverse events, the number of such events, the perceived impact of such events, and methylation variables). Missing confounders were imputed as the sample mean of the variable. Sub-analyses were also conducted to estimate the association between the exposures and the log-transformed CV measures at each age separately, and linear regression was used for these analyses instead of linear mixed models and adjusted for the minimal set of potential confounders. All estimates of associations for CV measures are for a 4-unit change in maternal adversities, which corresponds to the difference between the adversity not occurring and the adversity having its highest impact. Linear mixed models[16] were used to test the associations between individual CpG sites with



For peer review only - http://bmjopen.bmj.com/site/about/guidelines.xhtml

maternal adversity measures and child CV measures. The Bonferroni p-value threshold was used to correct for multiple testing in the analyses of individual methylation probes.

P-values were based on the likelihood ratio statistic except for the descriptive analyses, where p-values for a sex difference were based on a t-test (for continuous variables) or Fisher's exact test (for binary variables)[17]. All analyses were conducted in R version 4.0.0[18].

**Patient and public involvement**

There was no direct involvement from participants in the study design. Select participants are part of a committee which meets to discuss and provide insights on acceptability, and study methodology and design. This committee was not involved in the formulation of the current research question and analyses.

For peer review only



For peer review only - http://bmjopen.bmj.com/site/about/guidelines.xhtml

## Results

Characteristics and summary data of the sample are as shown in Table 1. The median number of maternal events and perceived impact score was 3.6 (2.3) and 8.5 (7), respectively (Table 1). The most common event during the study period was an argument with partner (63.1%), followed by foetal testing (52.6%) and reductions in income (50.6%) (Supplementary Table S1).

### Maternal adversity and overall offspring CV measures

There was no association between number of events and any of the primary offspring CV measures (CV time points combined) (Table 2). Results did not differ when analyses were re-run using perceived impact scores.

### Maternal adversity and offspring CV measures by sex and specific timepoints

In contrast to our hypotheses, there was an association between perceived impact score and PWV (time points combined) in boys, whereby a four-unit increase in adversity score was associated with a 0.1% decrease in PWV (0.999-fold change, 95% CI: 0.997, 1.001; Table 2). When HR and BP measures were examined at specific time points (i.e., 7-yrs and 17-yrs separately) there was an association between maternal number of events and offspring SBP at seven years of age in girls, whereby there was a 0.6% decrease in BP for each additional four events (0.994-fold change, 95% CI: 0.988, 0.999). In line with our hypotheses, there was also an association between offspring DBP at 17-years of age and maternal perceived impact score in girls whereby a four-unit increase in impact score was associated with a 0.2% increase in DBP (1.002-fold change 95% CI: 1.000, 1.005). There were no other associations detected with number of events or perceived impact score at specific time points (data not shown).



For peer review only - http://bmjopen.bmj.com/site/about/guidelines.xhtml

For peer review only

**DNA methylation and offspring CV measures**

In line with our hypotheses, there were some associations evident with specific CpG sites. In the longitudinal analyses, with timepoints combined, methylation of cg20111643 (TOM1L1) was associated with offspring SBP (1.013-fold change 95% CI: 1.008, 1.017 per standard deviation). There was an association with methylation of cg07494499 (NXN) (1.012-fold change 95% CI: 1.008, 1.017 per standard deviation of the outcome) and cg02458152 (EFCAB1) (1.011-fold change 95% CI: 1.007, 1.015 per standard deviation) and SBP. There was also an association between methylation of cg20222926 (FEZF1) (0.987-fold change 95% CI: 0.982, 0.992 per standard deviation) and DBP that appeared to be largely driven by rare, large DNA methylation changes (Figure 2). However, when the 3 outliers were excluded, the effect was no longer observed. There were no associations with any other CpG site.

**Specific adversities and offspring CV measures**

We further explored how events clustered (Supplementary Figure S3), and whether specific events were associated with offspring CV measures stratified by sex (Table 3).

Of the 43 maternal adversity events examined, 5 showed associations with slightly favourable CV health in the offspring at age 7 years. These associations differed by sex.

In contrast to the hypotheses, at the seven-year follow-up, mothers who had argued with their partner during pregnancy had female offspring with 2.4% lower HR (0.976 fold-change 95% CI: 0.961, 0.992); those who took an exam during pregnancy had female offspring with 4.2% lower SBP (0.958-fold-change 95% CI: 0.928, 0.989); and those admitted to hospital during pregnancy had female offspring with 2.5% lower DBP (0.975 fold-change 95% CI: 0.956, 0.992). Mothers who had an ill partner during pregnancy had male offspring with 2.9% lower SBP (0.971-fold change 95% CI: 0.951, 0.992); and those who become homeless had male offspring with 7.3% lower DBP (0.927-fold change 95% CI: 0.88, 0.978). However, in



For peer review only - http://bmjopen.bmj.com/site/about/guidelines.xhtml

agreement with the hypotheses, at 17-years, mothers whose partner rejected the pregnancy had female offspring with 7.2% higher HR (1.072-fold change 95% CI: 1.019, 1.127); and mothers who moved house during pregnancy had male offspring with 4.5% higher HR (1.045-fold change 95% CI: 1.012, 1.079). Mothers who were convicted of an offence or took an exam during pregnancy had male offspring with 91.9% (1.919-fold change 95% CI:1.209, 3.041) and 9.8% (1.098-fold change 95% CI: 1.027, 1.174) higher HR. Women who reported they were very ill during pregnancy had male offspring with 3.2% higher SBP (1.032-fold change 95%CI: 1.009, 1.056); and 4.2% DBP (1.042-fold change 95% CI: 1.03, 1.072), respectively. Contrasting the hypotheses, at 17 years of age mothers whose partner hurt the child during pregnancy had male offspring with 34.5% lower HR (0.655-fold change 95% CI: 0.492, 0.873).

For peer review only

## Discussion

There was no evidence of an overall association between our primary CV measures in offspring and maternal adversity. There was limited evidence to suggest that sub-types of adversity or specific CpG probes may be associated with CV measures in an age-specific manner.

Associations between adversity and health outcomes previously reported in the literature are thought to be moderated by biological changes induced by the stress response. Global methylation is associated with CVD in adult populations[19]. However, the association between epigenetic changes at birth and CV measures in childhood and adolescence is less well-characterised. It could be that infancy and childhood is a more sensitive period to CV changes induced by adversity than during pregnancy and, although previous results are mixed. For instance, there is support for associations between childhood maltreatment and CV disease and risk factors in adulthood[20]. It is possible that exposure to adversities experienced by this cohort were not severe, nor prolonged enough, to have a direct impact on DNA methylation and/or on cardiac function. There were some associations with specific CpG sites, cg20111643 (TOM1L1), cg07494499 (NXN), cg02458152 (EFCAB1) and cg20222926 (FEZF1). Of the genes that these sites are located on only one, NXN, has a postulated role in cardiac development through its role in the canonical Wnt/β-catenin signalling pathway[21]. Interestingly EFCAB1 has also been implicated in BP measurements[22], as was observed in this cohort. Of note is the association with cg20222926 (FEZF1), which may be the result of interesting biology, or could be a consequence of measurement error. Future investigations should also consider whether factors such as exposure to maternal hypertensive disorders in utero, such as pre-eclampsia, may play a role in the causal pathway of any observed associations.



For peer review only - http://bmjopen.bmj.com/site/about/guidelines.xhtml

Few studies have looked at maternal adversity and CV risk factors in childhood and adolescence. Within this cohort, no association was observed between childhood adversity and blood pressure at seven and 11 years of age[23]. In an Australian cohort of children those with lower psychosocial stress had higher pulse pressure at age 11[24]. This finding is similar to the favourable associations observed in our study between specific adversities and offspring CV measures, at 7 years of age. Given that this is a paediatric study population it is possible that the unexpected increases in BP observed at seven years of age may be a feature of the developing CV system in the offspring[25]. Of further consideration is that CV measures during childhood and adolescence may not wholly predict progression to CV disease in adulthood[26]. Thus, the results presented do not preclude further examination of perinatal adversity and CV disease and risk in adulthood. However, while these measures do not wholly predict progression during adulthood the observed associations between maternal adversity and offspring CV markers, such as BP and PWV, may be early evidence of cardiovascular dysfunction. It is plausible that the risk pathways between maternal stress and CVD risk are activated, but the full extent of damage is not yet evident. This would be consistent with the accumulation hypothesis of lifecourse epidemiology, which purports that health disparities become more pronounced with age (i.e diverge)[27]. Moreover, the measure of maternal adversity used in this study was an inventory of life events, not based on a conceptual framework, such as that of the adverse childhood experiences construct. Thus, this measure of adversity may not have captured all stressors during pregnancy, which may conceal a legitimate association and in part explain the null findings. Lastly, emerging evidence has suggested that the human placenta may buffer the effects of maternal stress and protect the developing fetus[5], which could provide a biological explanation for apparent absent effects of maternal stress in this cohort.



For peer review only - http://bmjopen.bmj.com/site/about/guidelines.xhtml

Specific adversities were associated with favourable changes in offspring CV measures at age seven. At age 17, the direction of the association largely reversed. This is suggestive of a protective adaptive response to maternal adversity present in childhood that may reverse trajectory by age 17. Contrary to the original hypotheses, at age seven, specific maternal adversities largely appeared to have a protective effect on offspring CV measures. Similarly, in this same cohort, a different study observed that maternal prenatal anxiety and depressive symptomology was inversely associated with offspring blood pressure at 10-11 years of age, albeit to a similar magnitude as paternal measures[28]. However, given this association was not looked at beyond 11 years of age it is not known if a similar reversal of trajectory was present at 17-years. Given multiple comparisons, it is also possible that the associations between specific adversities and offspring CV have arisen due to chance. However, it is curious that the associations largely follow the same age-trajectory, that is an inverse association with adversity events at seven years and a positive association at 17 years. It is also noteworthy that reported adversities that had the largest effect size were those that would presumably have more psychological impact e.g. partner hurt child and mother convicted of an offence. Nevertheless, replication in other cohorts would have to be demonstrated to confirm such associations.

A strength of the current study is its large sample size and its detailed collection of longitudinal phenotypic data in both mothers and their children followed into adolescence. However, as is the case with such long-term observational studies, over time, there is evidence of attrition, which may introduce bias, with those who were of a higher socioeconomic position being more likely to remain in the study over time thus potentially limiting the generalisabilty of the results. Moreover, the list of potentially life stressors was not exhaustive and may have resulted in measurement error influencing the results. In addition, the adversity scores calculated as part of this study have not been previously validated. Furthermore, to capture maternal adversity



For peer review only - http://bmjopen.bmj.com/site/about/guidelines.xhtml

during pregnancy, we took the weighted average of the Life Events Inventory, which was inclusive from the beginning of pregnancy to 8 weeks post-partum). Thus, any effects may have been diluted by the inclusion of adversity in the eight weeks post birth during the perinatal period. Lastly, future studies may benefit from the examination of specific key genes that have been identified in CVD pathways aside from global methylation measures.

In summary, the results presented largely do not support an association between maternal prenatal adversity, and offspring methylation and CV measures during childhood and adolescence. There were, however some sex- and age- specific trends which would have to be confirmed in future studies. Identification and confirmation of these associations between maternal adversity and offspring cardiovascular function may assist with identifying high risk populations for which additional monitoring may be appropriate.

**Funding**

The UK Medical Research Council and Wellcome (Grant ref: 217065/Z/19/Z) and the University of Bristol provide core support for ALSPAC. A comprehensive list of grants funding is available on the ALSPAC website (http://www.bristol.ac.uk/alspac/external/documents/grant-acknowledgements.pdf); This research was specifically funded by the British Heart Foundation who provided support for the collection of pulse wave velocity measures (RG/10/004/28240GWAS). Data was generated by Sample Logistics and Genotyping Facilities at Wellcome Sanger Institute and LabCorp (Laboratory Corporation of America) using support from 23andMe. This specific analyses were supported by a grant from the University of Melbourne.

**Competing interests**

None declared.

For peer review only

### Contributors

AO conceived the initial idea for examining the associations and all authors made a substantial contribution to the conception and/or design of the study analyses. JGD performed all statistical analyses. NKH wrote the initial draft of the manuscript and all authors reviewed (NKH, JGD, AS, GA, GS, LO, KL and AO) and contributed intellectual content. All authors have approved of the final version that has been submitted.

### Data availability statement

Please note that the study website contains details of all the data that is available through a fully searchable data dictionary and variable search tool (http://www.bristol.ac.uk/alspac/researchers/our-data/.) The data underlying this article will be shared on reasonable request to the corresponding author with permission from the ALSPAC team in accordance with data sharing agreements.

### Ethical approval

Ethical approval for the study was obtained from the ALSPAC Ethics and Law Committee and the Local Research Ethics Committees and The University of Melbourne Human Research Ethics committee (ref: 1853268.1). Consent for biological samples has been collected in accordance with the Human Tissue Act (2004) and informed consent for the use of data collected via questionnaires and clinics was obtained from participants following the recommendations of the ALSPAC Ethics and Law Committee at the time.



For peer review only - http://bmjopen.bmj.com/site/about/guidelines.xhtml

## References

1. Barker DJ, Osmond C, Law CM. The intrauterine and early postnatal origins of cardiovascular disease and chronic bronchitis. *Journal of Epidemiology and Community Health* 1989;43(3):237-40. doi: 10.1136/jech.43.3.237
2. Barker DJ, Osmond C, Golding J, et al. Growth in utero, blood pressure in childhood and adult life, and mortality from cardiovascular disease. *British Medical Journal* 1989;298(6673):564-67. doi: 10.1136/bmj.298.6673.564
3. Gillman MW, Barker D, Bier D, et al. Meeting report on the 3rd International Congress on Developmental Origins of Health and Disease (DOHaD). *Pediatric research* 2007;61(5 Pt 1):625-9. doi: 10.1203/pdr.0b013e3180459fcd [published Online First: 2007/04/07]
4. Sosnowski DW, Booth C, York TP, et al. Maternal prenatal stress and infant DNA methylation: A systematic review. *Developmental psychobiology* 2018;60(2):127-39. doi: 10.1002/dev.21604 [published Online First: 2018/01/19]
5. Vuppaladhadiam L, Lager J, Fiehn O, et al. Human Placenta Buffers the Fetus from Adverse Effects of Perceived Maternal Stress. *Cells* 2021;10(2):379.
6. Gilbert JS. Chapter 8 - Epigenetics in the Developmental Origin of Cardiovascular Disorders. In: Rosenfeld CS, ed. The Epigenome and Developmental Origins of Health and Disease. Boston: Academic Press 2016:127-41.
7. Suglia SF, Koenen KC, Boynton-Jarrett R, et al. Childhood and Adolescent Adversity and Cardiometabolic Outcomes: A Scientific Statement From the American Heart Association. *Circulation* 2018;137(5):e15-e28. doi: 10.1161/cir.0000000000000536 [published Online First: 2017/12/20]
8. Boyd A, Golding J, Macleod J, et al. Cohort Profile: The 'Children of the 90s'—the index offspring of the Avon Longitudinal Study of Parents and Children. *International Journal of Epidemiology* 2012;42(1):111-27. doi: 10.1093/ije/dys064
9. Fraser A, Macdonald-Wallis C, Tilling K, et al. Cohort Profile: The Avon Longitudinal Study of Parents and Children: ALSPAC mothers cohort. *International Journal of Epidemiology* 2012;42(1):97-110. doi: 10.1093/ije/dys066
10. Barnett BE, Hanna B, Parker G. Life event scales for obstetric groups. *Journal of psychosomatic research* 1983;27(4):313-20. doi: 10.1016/0022-3999(83)90054-5 [published Online First: 1983/01/01]
11. O'Keeffe LM, Howe LD, Fraser A, et al. Associations of Y chromosomal haplogroups with cardiometabolic risk factors and subclinical vascular measures in males during childhood and adolescence. *Atherosclerosis* 2018;274:94-103. doi: 10.1016/j.atherosclerosis.2018.04.027 [published Online First: 2018/05/13]
12. Relton CL, Gaunt T, McArdle W, et al. Data Resource Profile: Accessible Resource for Integrated Epigenomic Studies (ARIES). *Int J Epidemiol* 2015;44(4):1181-90. doi: 10.1093/ije/dyv072 [published Online First: 2015/05/21]
13. Richmond RC, Simpkin AJ, Woodward G, et al. Prenatal exposure to maternal smoking and offspring DNA methylation across the lifecourse: findings from the Avon Longitudinal Study of Parents and Children (ALSPAC). *Human molecular genetics* 2015;24(8):2201-17. doi: 10.1093/hmg/ddu739 [published Online First: 2015/01/02]
14. Aryee MJ, Jaffe AE, Corrada-Bravo H, et al. Minfi: a flexible and comprehensive Bioconductor package for the analysis of Infinium DNA methylation microarrays. *Bioinformatics (Oxford, England)* 2014;30(10):1363-9. doi: 10.1093/bioinformatics/btu049 [published Online First: 2014/01/31]
15. Houseman EA, Accomando WP, Koestler DC, et al. DNA methylation arrays as surrogate measures of cell mixture distribution. *BMC Bioinformatics* 2012;13(1):86. doi: 10.1186/1471-2105-13-86



For peer review only - http://bmjopen.bmj.com/site/about/guidelines.xhtml

16. Schall R. Estimation in Generalized Linear Models with Random Effects. *Biometrika* 1991;78(4):719-27. doi: 10.2307/2336923
17. Agresti A. Categorical data analysis: John Wiley & Sons 2003.
18. Team RC. R: A language and environment for statistical computing: Vienna, Austria, 2013.
19. Muka T, Koromani F, Portilla E, et al. The role of epigenetic modifications in cardiovascular disease: A systematic review. *International journal of cardiology* 2016;212:174-83. doi: 10.1016/j.ijcard.2016.03.062 [published Online First: 2016/04/04]
20. Basu A, McLaughlin KA, Misra S, et al. Childhood Maltreatment and Health Impact: The Examples of Cardiovascular Disease and Type 2 Diabetes Mellitus in Adults. *Clinical psychology : a publication of the Division of Clinical Psychology of the American Psychological Association* 2017;24(2):125-39. doi: 10.1111/cpsp.12191 [published Online First: 2017/09/05]
21. Mak CCY, Chow PC, Liu APY, et al. De novo large rare copy-number variations contribute to conotruncal heart disease in Chinese patients. *npj Genomic Medicine* 2016;1(1):16033. doi: 10.1038/npjgenmed.2016.33
22. Ho JE, Levy D, Rose L, et al. Discovery and replication of novel blood pressure genetic loci in the Women's Genome Health Study. *Journal of hypertension* 2011;29(1):62-9. doi: 10.1097/HJH.0b013e3283406927 [published Online First: 2010/11/04]
23. Slopen N, Koenen KC, Kubzansky LD. Cumulative adversity in childhood and emergent risk factors for long-term health. *The Journal of pediatrics* 2014;164(3):631-8.e1-2. doi: 10.1016/j.jpeds.2013.11.003 [published Online First: 2013/12/19]
24. Olive LS, Abhayaratna WP, Byrne D, et al. Depression, stress and vascular function from childhood to adolescence: A longitudinal investigation. *General Hospital Psychiatry* 2020;62:6-12. doi: https://doi.org/10.1016/j.genhosppsych.2019.10.001
25. Wilkinson IB, Franklin SS, Hall IR, et al. Pressure amplification explains why pulse pressure is unrelated to risk in young subjects. *Hypertension (Dallas, Tex : 1979)* 2001;38(6):1461-6. doi: 10.1161/hy1201.097723 [published Online First: 2001/12/26]
26. Urbina EM, Khoury PR, Bazzano L, et al. Relation of Blood Pressure in Childhood to Self-Reported Hypertension in Adulthood. *Hypertension (Dallas, Tex : 1979)* 2019;73(6):1224-30. doi: 10.1161/hypertensionaha.118.12334 [published Online First: 2019/05/09]
27. Dannefer D. Cumulative Advantage/Disadvantage and the Life Course: Cross-Fertilizing Age and Social Science Theory. *The Journals of Gerontology: Series B* 2003;58(6):S327-S37. doi: 10.1093/geronb/58.6.S327
28. van Dijk AE, Dawe K, Deanfield J, et al. The association of maternal prenatal psychosocial stress with vascular function in the child at age 10-11 years: findings from the Avon longitudinal study of parents and children. *Eur J Prev Cardiol* 2014;21(9):1097-108. doi: 10.1177/2047487313486039 [published Online First: 2013/04/04]

For peer review only



For peer review only - http://bmjopen.bmj.com/site/about/guidelines.xhtml

**Table 1:** Participant characteristics at each follow-up

| | *n* | Pooled sample Mean (±SD)/Median (IQR)/n (%) | Boys Mean (±SD)/Median (IQR)/n (%) | Girls Mean (±SD)/Median (IQR)/n (%) | p for sex difference |
|---|---|---|---|---|---|
| **Pregnancy and birth measures (n=14,901)** | | | | | |
| Maternal age (years at birth) | 12921 | 28 (5) | 28.1 (5) | 27.9 (4.9) | 0.009 |
| Maternal smoking status n (%) yes | 11052 | 2157 (19.5%) | 1144 (20.2%) | 1013 (18.8%) | 0.08 |
| Gestation length (weeks) | 12921 | 39.8 (1.3) | 39.7 (1.3) | 39.8 (1.3) | <0.001 |
| Number of events | 12285 | 3.6 (2.3) | 3.6 (2.3) | 3.6 (2.3) | 0.4 |
| Perceived impact score | 12285 | 8.5 (7) | 8.4 (7) | 8.6 (7.1) | 0.2 |
| Birthweight (g) | 12766 | 3469 (478) | 3530 (490) | 3404 (457) | <0.001 |
| Breastfed (% yes) | 10359 | 6185 (59.7%) | 3132 (59%) | 3053 (60.5%) | 0.1 |
| **Offspring 7-year follow-up (n=7431)** | | | | | |
| Systolic BP | 7065 | 98.8 (9.2) | 98.7 (9.1) | 98.9 (9.3) | 0.4 |
| Diastolic BP | 7063 | 56.5 (6.7) | 56.1 (6.7) | 56.9 (6.6) | <0.001 |
| HR | 7062 | 83.3 (10.7) | 82 (10.5) | 84.6 (10.8) | <0.001 |
| **Offspring 17-year follow-up (n=5,215)** | | | | | |
| Systolic BP | 4104 | 116.4 (9.9) | 122 (9.2) | 112 (8.1) | <0.001 |
| Diastolic BP | 4104 | 64.2 (6) | 63.3 (6) | 64.9 (5.9) | <0.001 |
| HR | 4104 | 65.2 (9.7) | 62.5 (9.2) | 67.2 (9.6) | <0.001 |
| cIMT | 4102 | 0.48 (0.05) | 0.48 (0.05) | 0.47 (0.04) | <0.001 |
| PWV | 3423 | 5.8 (0.7) | 6 (0.7) | 5.6 (0.6) | <0.001 |

*SES=socioeconomic status, BP=Blood pressure (mmHg), cIMT=carotid intermedia thickness, HR= heart rate (beat per minute), PWV= Pulse wave velocity*

*Maternal smoking is yes/no smoked cigarettes regularly in the last 2 months of pregnancy*

*Breastfeeding is yes/no 1+ months of breastfeeding*

For peer review only



For peer review only - http://bmjopen.bmj.com/site/about/guidelines.xhtml

**Table 2:** Associations between maternal adversity and offspring CV measures

| | | *Pooled* | *Boys* | *Girls* |
|---|---|---|---|---|
| **Outcome** | **Exposure** | **Fold change (95% CI)** | **Fold change (95% CI)** | **Fold change (95% CI)** |
| Resting heart rate (bpm) | *Perceived impact score* | 0.999 (0.998-1.001) | 1.000 (0.997-1.002) | 0.999 (0.997-1.001) |
| | *Adversity number* | 0.997 (0.992-1.003) | 0.998 (0.990-1.005) | 0.996 (0.989-1.004) |
| Systolic blood pressure (mmHg) | *Perceived impact score* | 1.000 (0.999-1.001) | 1.000 (0.998-1.002) | 1.000 (0.998-1.001) |
| | *Adversity number* | 0.998 (0.994-1.001) | 0.998 (0.993-1.003) | 0.998 (0.993-1.002) |
| Diastolic blood pressure (mmHg) | *Perceived impact score* | 1.000 (0.999-1.002) | 0.999 (0.997-1.002) | 1.001 (0.999-1.003) |
| | *Adversity number* | 0.999 (0.994-1.003) | 0.996 (0.989-1.002) | 1.001 (0.996-1.007) |
| Pulse-wave velocity | *Perceived impact score* | 0.999 (0.997-1.001) | 0.999 (0.997-1.001) | 1.001 (0.998-1.004) |
| | *Adversity number* | 0.998 (0.991-1.005) | 0.9928 (0.982-1.004) | 1.001 (0.992-1.010) |
| Carotid Intima Media Thickness | *Perceived impact score* | 1.000 (0.998-1.002) | 1.001 (0.998-1.004) | 1.000 (0.997-1.002) |
| | *Adversity number* | 1.001 (0.996-1.007) | 1.001 (0.993-1.010) | 1.001 (0.994-1.008) |

Models adjusted for child age, alcohol use in pregnancy; tobacco use in pregnancy; ethnic group; parity; age at delivery; and maternal education.

NB: Pulse wave velocity and Carotid Intima Media Thickness were only measured at one time point (17 years of age)**.** Fold changes corresponds to a 4 unit change in adversity measures.

For peer review only



For peer review only - http://bmjopen.bmj.com/site/about/guidelines.xhtml

## Table 3: Specific maternal adversities and longitudinal offspring CV measures

| *Adversity event* | *Heart rate* | | | *Systolic blood pressure* | | | *Diastolic blood pressure* | | |
|---|---|---|---|---|---|---|---|---|---|
| | **Pooled** | **Boys** | **Girl** | **Pooled** | **Boys** | **Girls** | **Pooled** | **Boys** | **Girls** |
| | **Fold change (95% CI)** | **Fold change (95% CI)** | **Fold change (95% CI)** | **Fold change (95% CI)** | **Fold change (95% CI)** | **Fold change (95% CI)** | **Fold change (95% CI)** | **Fold change (95% CI)** | **Fold change (95% CI)** |
| Partner died | 0.93 (0.80-1.09) | 0.93 (0.76-1.15) | 0.94 (0.74-1.18) | 0.98 (0.90-1.07) | 0.93 (0.83-1.05) | 1.04 (0.90-1.19) | 0.99 (0.89-1.11) | 0.95 (0.82-1.10) | 1.06 (0.90-1.26) |
| Child died | 1.06 (0.85-1.33) | 1.06 (0.71-1.57) | 1.06 (0.81-1.40) | 1.10 (0.94-1.27) | 1.08 (0.82-1.41) | 1.11 (0.94-1.33) | 1.15 (0.96-1.38) | 1.18 (0.84-1.67) | 1.14 (0.92-1.41) |
| Friend or relative died | 1.00 (0.99-1.02) | 1.01 (0.99-1.04) | 0.99 (0.97-1.02) | 0.99 (0.98-1.00) | 1.00 (0.98-1.01) | 0.99 (0.98-1.00) | 1.00 (0.99-1.01) | 1.00 (0.98-1.01) | 1.00 (0.99-1.02) |
| Child was ill | 0.99 (0.98-1.01) | 0.99 (0.98-1.02) | 0.99 (0.97-1.01) | 1.00 (0.99-1.01) | 1.00 (0.99-1.02) | 0.99 (0.98-1.00) | 1.00 (0.98-1.01) | 1.00 (0.98-1.02) | 0.99 (0.98-1.01) |
| Partner was ill | 1.00 (0.98-1.02) | 1.00 (0.97-1.03) | 1.00 (0.97-1.03) | 0.99 (0.98-1.00) | 0.98 (0.97-1.00) | 1.00 (0.98-1.02) | 1.00 (0.98-1.01) | 0.99 (0.96-1.01) | 1.01 (0.99-1.03) |
| Friend or relative was ill | 1.00 (0.98-1.01) | 1.01 (0.99-1.03) | 0.99 (0.97-1.01) | 1.00 (0.99-1.01) | 1.00 (0.99-1.01) | 1.00 (0.99-1.01) | 1.01 (0.99-1.02) | 1.01 (0.99-1.02) | 1.00 (0.99-1.02) |
| Admitted to hospital | 0.99 (0.98-1.01) | 1.01 (0.99-1.03) | 0.97 (0.96-0.99)[#] | 1.00 (0.99-1.00) | 1.00 (0.99-1.02) | 0.99 (0.98-1.00)[#] | 1.00 (0.99-1.01) | 1.00 (0.99-1.02) | 0.99 (0.97-1.01) |
| In trouble with the law | 1.02 (0.93-1.11) | 1.08 (0.95-1.24) | 0.97 (0.86-1.09) | 1.00 (0.96-1.05) | 0.91 (0.84-0.99)[#] | 1.06 (1.00-1.12) | 1.01 (0.95-1.07) | 0.97 (0.88-1.07) | 1.04 (0.97-1.11) |
| Divorced | 1.02 (0.95-1.10) | 1.05 (0.96-1.15) | 0.99 (0.89-1.10) | 1.04 (1.00-1.08) | 1.03 (0.97-1.09) | 1.06 (1.00-1.12) | 1.05 (0.99-1.10) | 1.04 (0.97-1.11) | 1.05 (0.98-1.13) |
| Partner rejected pregnancy | 1.00 (0.97-1.03) | 1.01 (0.97-1.05) | 1.00 (0.96-1.03) | 0.99 (0.98-1.01) | 0.98 (0.96-1.00) | 1.01 (0.99-1.03) | 1.01 (0.99-1.03) | 0.99 (0.97-1.02) | 1.02 (1.00-1.05) |
| Very ill | 1.00 (0.98-1.02) | 1.02 (0.99-1.04) | 1.00 (0.98-1.03) | 1.01 (1.00-1.02) | 1.01 (0.99-1.03) | 1.01 (0.99-1.03) | 1.02 (1.01-1.04)[*] | 1.03 (1.01-1.05)[*] | 1.01 (0.99-1.03) |
| Partner lost job | 1.00 (0.98-1.01) | 1.00 (0.97-1.03) | 0.99 (0.97-1.02) | 1.00 (0.99-1.01) | 1.01 (0.99-1.02) | 1.00 (0.98-1.02) | 1.01 (1.00-1.03) | 1.01 (0.99-1.04) | 1.01 (0.99-1.03) |
| Partner had problems at work | 0.99 (0.98-1.01) | 0.99 (0.97-1.01) | 1.00 (0.98-1.01) | 1.00 (0.99-1.01) | 1.00 (0.99-1.01) | 1.00 (0.99-1.01) | 1.00 (0.99-1.01) | 0.99 (0.97-1.00) | 1.00 (0.99-1.01) |
| Problems at work | 1.00 (0.98-1.01) | 0.99 (0.98-1.02) | 1.00 (0.97-1.02) | 1.01 (1.00-1.02) | 1.01 (0.99-1.03) | 1.01 (0.99-1.02) | 1.01 (1.00-1.02) | 1.01 (0.99-1.03) | 1.02 (1.00-1.03) |
| Lost job | 0.99 (0.96-1.02) | 0.97 (0.93-1.02) | 1.00 (0.96-1.04) | 1.00 (0.98-1.02) | 1.01 (0087-1.04) | 1.00 (0.98-1.03) | 1.01 (0.99-1.03) | 1.01 (0.98-1.05) | 1.01 (0.98-1.04) |
| Partner went away | 1.00 (0.98-1.02) | 1.00 (0.97-1.02) | 1.00 (0.98-1.02) | 1.00 (0.99-1.01) | 0.99 (0.98-1.01) | 1.00 (0.99-1.02) | 1.00 (0.98-1.01) | 0.99 (0.97-1.01) | 1.00 (0.99-1.02) |
| Partner in trouble with law | 0.98 (0.95-1.02) | 1.00 (0.95-1.06) | 0.97 (0.92-1.02) | 1.00 (0.98-1.03) | 0.99 (0.95-1.03) | 1.02 (0.99-1.06) | 0.98 (0.95-1.01) | 0.95 (0.91-1.00)[#] | 1.00 (0.96-1.04) |
| Separated | 1.01 (0.99-1.03) | 1.01 (0.98-1.04) | 1.01 (0.98-1.04) | 1.01 (0.99-1.02) | 0.99 (0.97-1.01) | 1.03 (1.01-1.04)[*] | 1.00 (0.98-1.02) | 0.99 (0.96-1.02) | 1.01 (0.99-1.04) |
| Income reduced | 1.00 (0.99-1.01) | 1.01 (0.99-1.02) | 0.99 (0.98-1.00) | 1.00 (1.00-1.01) | 1.01 (1.00-1.02)[#] | 1.00 (0.99-1.01) | 1.01 (1.00-1.02) | 1.01 (1.00-1.03) | 1.01 (0.99-1.02) |
| Argued with partner | 0.99 (0.98-1.00) | 1.00 (0.99-1.02) | 0.98 (0.97-1.00 [#] | 1.00 (0.99-1.00) | 1.00 (0.99-1.01) | 0.99 (0.98-1.00) | 1.00 (0.99-1.01) | 1.00 (0.99-1.01) | 0.99 (0.98-1.00) |
| Argued with family or friends | 1.00 (0.98-1.01) | 1.00 (0.98-1.02) | 1.00 (0.98-1.02) | 1.00 (0.99-1.01) | 1.00 (0.99-1.02) | 0.99 (0.98-1.00) | 1.00 (0.99-1.01) | 1.00 (0.98-1.01) | 1.00 (0.99-1.02) |
| Moved house | 1.02 (1.00-1.03) | 1.01 (0.99-1.04) | 1.02 (1.00-1.04)[#] | 1.00 (0.99-1.01) | 1.00 (0.99-1.02) | 1.00 (0.99-1.01) | 0.99 (0.98-1.01) | 0.99 (0.97-1.01) | 1.00 (0.98-1.01) |
| Partner hurt mother | 0.99 (0.95-1.03) | 0.99 (0.94-1.05) | 0.99 (0.94-1.04) | 0.99 (0.97-1.02) | 0.98 (0.95-1.02) | 1.01 (0.98-1.04) | 0.97 (0.94-1.00)[#] | 0.95 (0.90-0.99)[#] | 0.98 (0.94-1.02) |

For peer review only

| | | | | | | | | | |
|---|---|---|---|---|---|---|---|---|---|
| Became homeless | 1.00 (0.97-1.04) | 1.00 (0.95-1.06) | 1.00 (0.95-1.05) | 1.00 (0.98-1.03) | 0.99 (0.96-1.03) | 1.01 (0.98-1.05) | 0.97 (0.94-1.00)# | 0.94 (0.90-0.98)* | 1.00 (0.96-1.04) |
| Major financial problems | 1.01 (1.00-1.02) | 1.01 (0.99-1.03) | 1.01 (0.99-1.02) | 1.01 (1.00-1.02) | 1.01 (1.00-1.02) | 1.01 (1.00-1.02) | 1.01 (1.00-1.02) | 1.01 (0.99-1.02) | 1.01 (1.00-1.03)# |
| Got married | 1.00 (0.97-1.03) | 0.98 (0.94-1.03) | 1.02 (0.97-1.07) | 1.00 (0.97-1.01) | 1.00 (0.97-1.02) | 0.99 (0.96-1.02) | 1.00 (0.98-1.03) | 1.00 (0.96-1.03) | 1.01 (0.97-1.05) |
| Partner hurt child | 0.94 (0.82-1.08) | 0.73 (0.59-0.91)^ | 1.11 (0.94-1.32) | 1.03 (0.94-1.11) | 0.93 (0.81-1.07) | 1.06 (0.96-1.18) | 0.95 (0.85-1.05) | 0.87 (0.73-1.04) | 1.00 (0.88-1.14) |
| Attempted suicide | 1.02 (1.05-1.39)* | 1.26 (1.02 -1.56)# | 1.16 (0.95-1.40) | 0.97 (0.88-1.06) | 1.01 (0.88-1.16) | 0.96 (0.85-1.08) | 1.07 (0.96-1.21) | 1.09 (0.92-1.31) | 1.06 (0.91-1.23) |
| Convicted of an offence | 1.09 (0.97-1.23) | 1.14 (0.93-1.39) | 1.07 (0.93-1.23) | 0.99 (0.92-1.07) | 0.86 (0.75-0.98)# | 1.07 (0.97-1.17) | 1.02 (0.93-1.12) | 0.98 (0.83-1.15) | 1.04 (0.94-1.17) |
| Bled & thought might miscarry | 0.99 (0.97-1.00) | 0.99 (0.97-1.01) | 0.99 (0.97-1.01) | 1.00 (1.00-1.01) | 1.00 (0.99-1.02) | 1.00 (0.99-1.02) | 1.00 (0.99-1.01) | 1.00 (0.98-1.01) | 1.00 (0.99-1.02) |
| Started new job | 1.00 (0.97-1.04) | 0.99 (0.94-1.04) | 1.01 (0.97-1.06) | 0.99 (0.97-1.02) | 0.99 (0.95-1.02) | 0.99 (0.96-1.02) | 1.00 (0.99-1.01) | 0.99 (0.97-1.00) | 1.00 (0.99-1.01) |
| Test to see if baby abnormal | 1.00 (0.98-1.01) | 0.99 (0.97-1.01) | 1.00 (0.98-1.02) | 1.00 (0.99-1.01) | 0.99 (0.98-1.00) | 1.00 (0.99-1.01) | 1.00 (0.99-1.01) | 0.99 (0.97-1.00) | 1.00 (0.99-1.02) |
| Tests show baby possibly abnormal | 1.00 (0.97-1.02) | 0.99 (0.96-1.02) | 1.01 (0.98-1.04) | 1.00 (0.98-1.01) | 0.99 (0.96-1.01) | 1.01 (0.99-1.03) | 1.00 (0.98-1.02) | 0.99 (0.96-1.01) | 1.01 (0.98-1.04) |
| Told having twins | 1.05 (1.00-1.11) | 1.03 (0.95-1.10) | 1.09 (1.00-1.19)# | 0.99 (0.96-1.03) | 0.99 (0.95-1.04) | 0.99 (0.94-1.04) | 0.98 (0.94-1.02) | 0.97 (0.92-1.03) | 0.99 (0.92-1.06) |
| Possible harm to baby | 0.99 (0.97-1.01) | 0.98 (0.95-1.00) | 1.00 (0.97-1.02) | 1.00 (0.99-1.02) | 1.00 (0.98-1.01) | 1.01 (0.99-1.03) | 1.00 (0.99-1.02) | 0.99 (0.97-1.02) | 1.02 (0.99-1.04) |
| Tried to have abortion | 1.04 (0.98-1.10) | 1.04 (0.96-1.14) | 1.03 (0.95-1.12) | 1.01 (0.98-1.06) | 0.99 (0.94-1.05) | 1.04 (0.98-1.09) | 1.02 (0.98-1.08) | 1.03 (0.96-1.11) | 1.03 (0.96-1.10) |
| Took an exam | 1.01 (0.99-1.04) | 1.04 (1.00-1.09) | 0.99 (0.95-1.03)# | 0.99 (0.97-1.01) | 1.01 (0.98-1.04) | 0.97 (0.94-0.99)* | 0.99 (0.97-1.01) | 1.01 (0.97-1.04) | 0.98 (0.94-1.01) |
| Partner emotionally cruel to mother | 0.99 (0.97-1.01) | 0.98 (0.96-1.00) | 1.00 (0.97-1.02) | 1.00 (0.99-1.02) | 1.01 (0.99-1.02) | 1.00 (0.98-1.02) | 1.01 (0.99-1.02) | 1.00 (0.91-1.02) | 1.01 (0.99-1.03) |
| Partner emotionally cruel to child | 0.99 (0.95-1.04) | 1.01 (0.93-1.09) | 0.98 (0.91-1.05) | 0.99 (0.96-1.02) | 0.98 (0.93-1.03) | 0.99 (0.96-1.04) | 0.97 (0.94-1.01) | 1.01 (0.94-1.08) | 0.96 (0.91-1.00) |
| House or car burgled | 1.00 (0.97-1.01) | 0.98 (0.95-1.01) | 1.00 (0.97-1.03) | 0.99 (0.97-1.00) | 1.00 (0.97-1.02) | 0.99 (0.97-1.01) | 0.99 (0.98-1.01) | 0.98 (0.96-1.01) | 1.00 (0.97-1.02) |
| Had an accident | 1.01 (0.92-1.05) | 1.00 (0.95-1.04) | 1.03 (1.00-1.08) | 0.99 (0.97-1.01) | 0.98 (0.96-1.01) | 0.99 (0.96-1.02) | 1.00 (0.97-1.02) | 0.99 (0.96-1.03) | 1.00 (0.96-1.03) |

# denotes p≤0.05

* denotes p≤0.01

^ denotes p≤0.001

**NB:** Fold changes represent a 4 unit change.

For peer review only

**Figure 1**: Participation flowchart

**Figure 2:** The relationship between CpG probe cg20222926 and offspring diastolic blood pressure

For peer review only



For peer review only - http://bmjopen.bmj.com/site/about/guidelines.xhtml

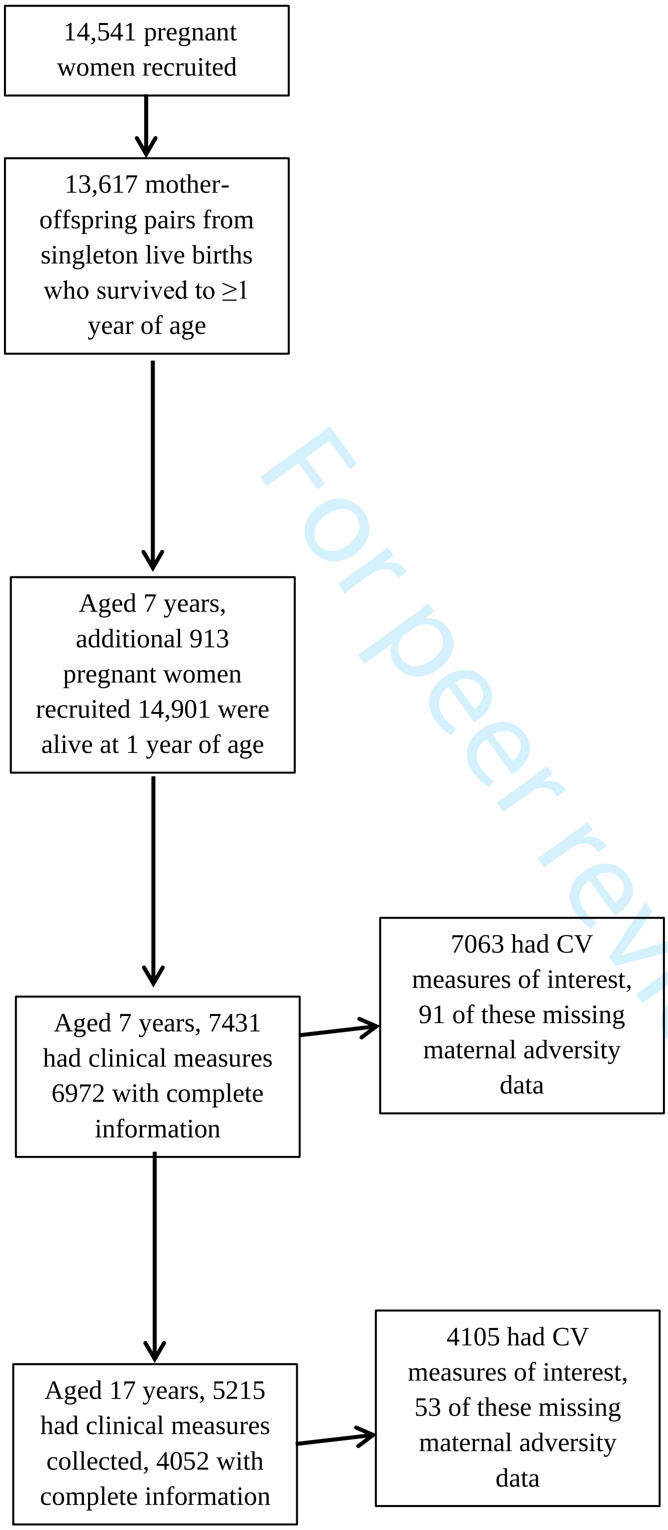


**Figure 1**: Participation flowchart

For peer review only

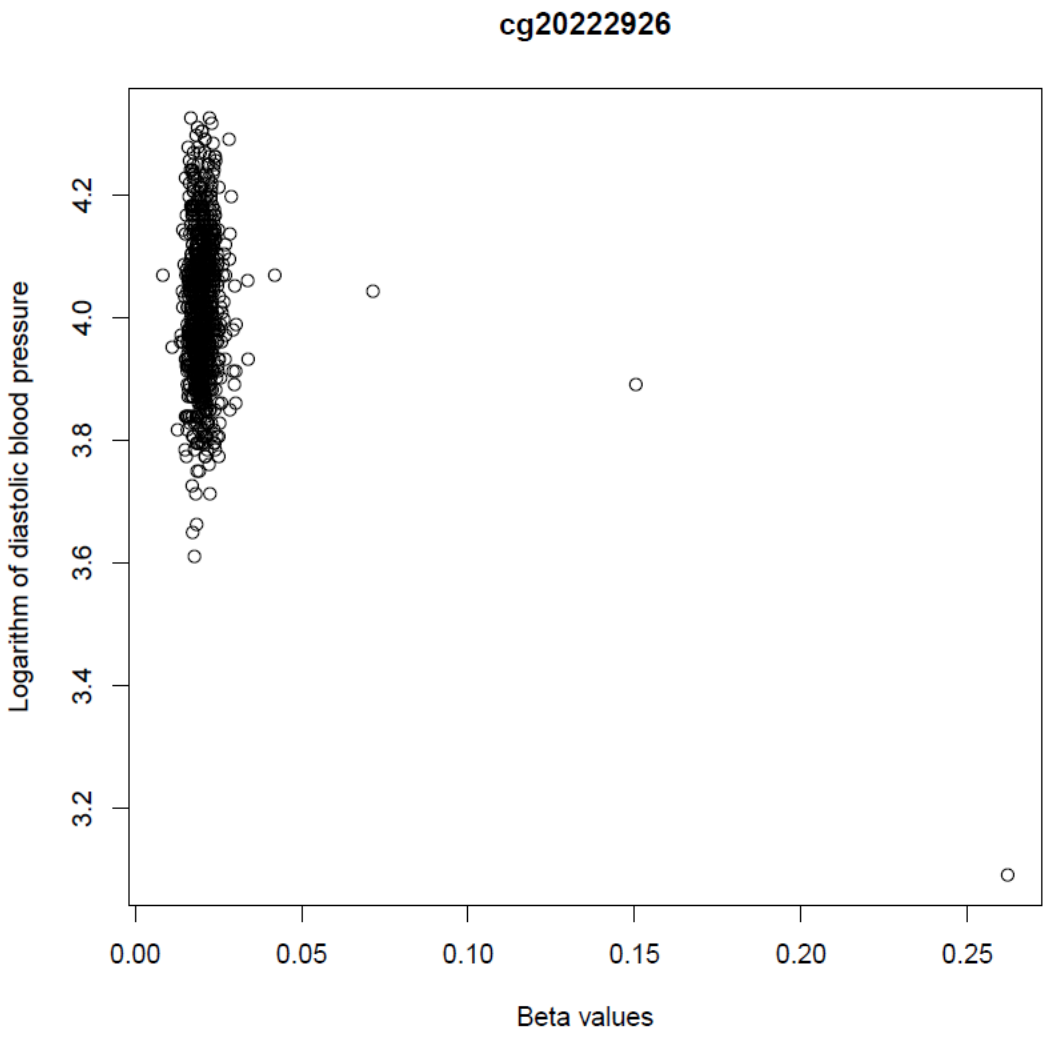


**Figure 2:** The relationship between CpG probe cg20222926 and offspring diastolic blood pressure

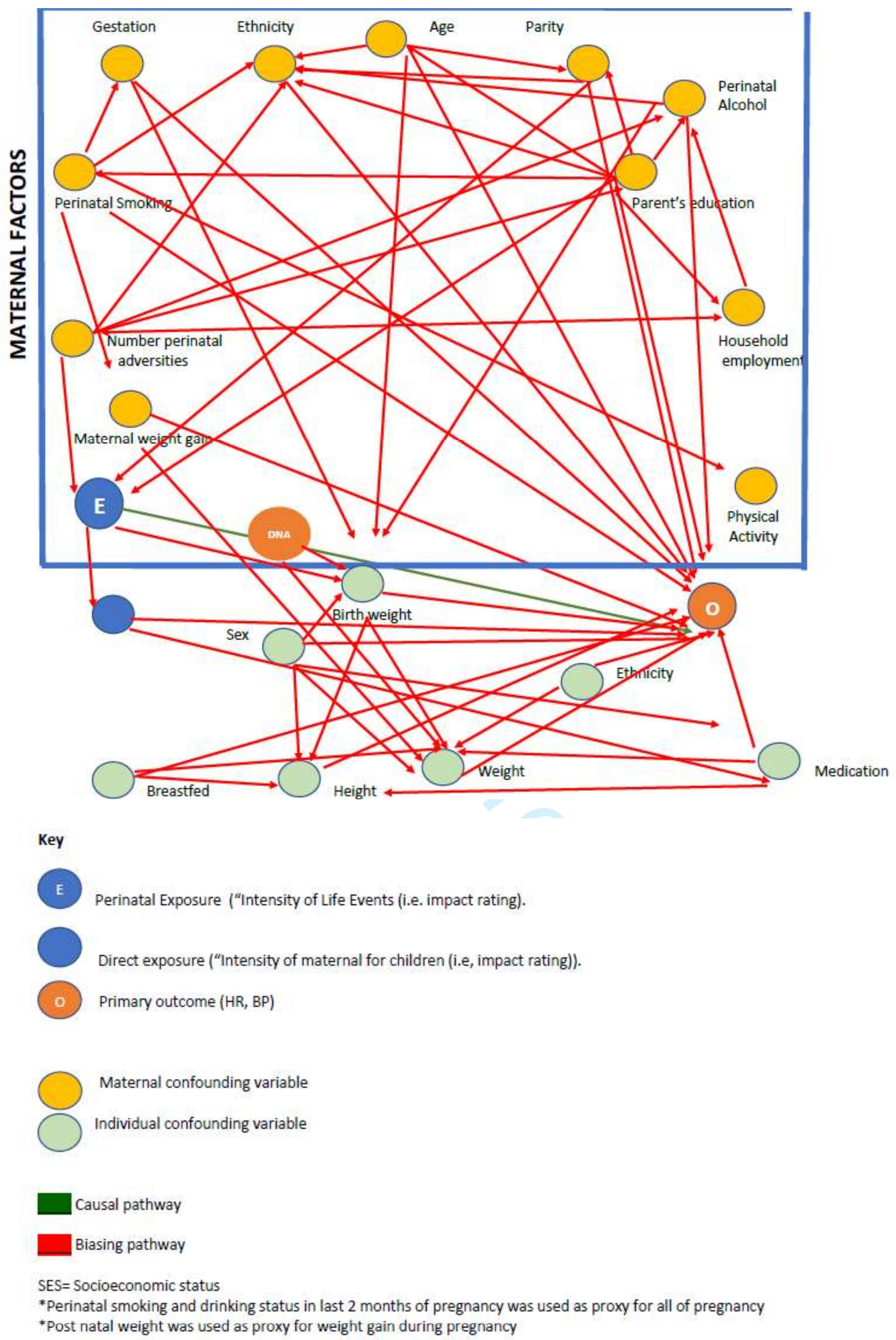


**Supplementary Figure S1:** Directed Acyclic Graph at 7 years of age

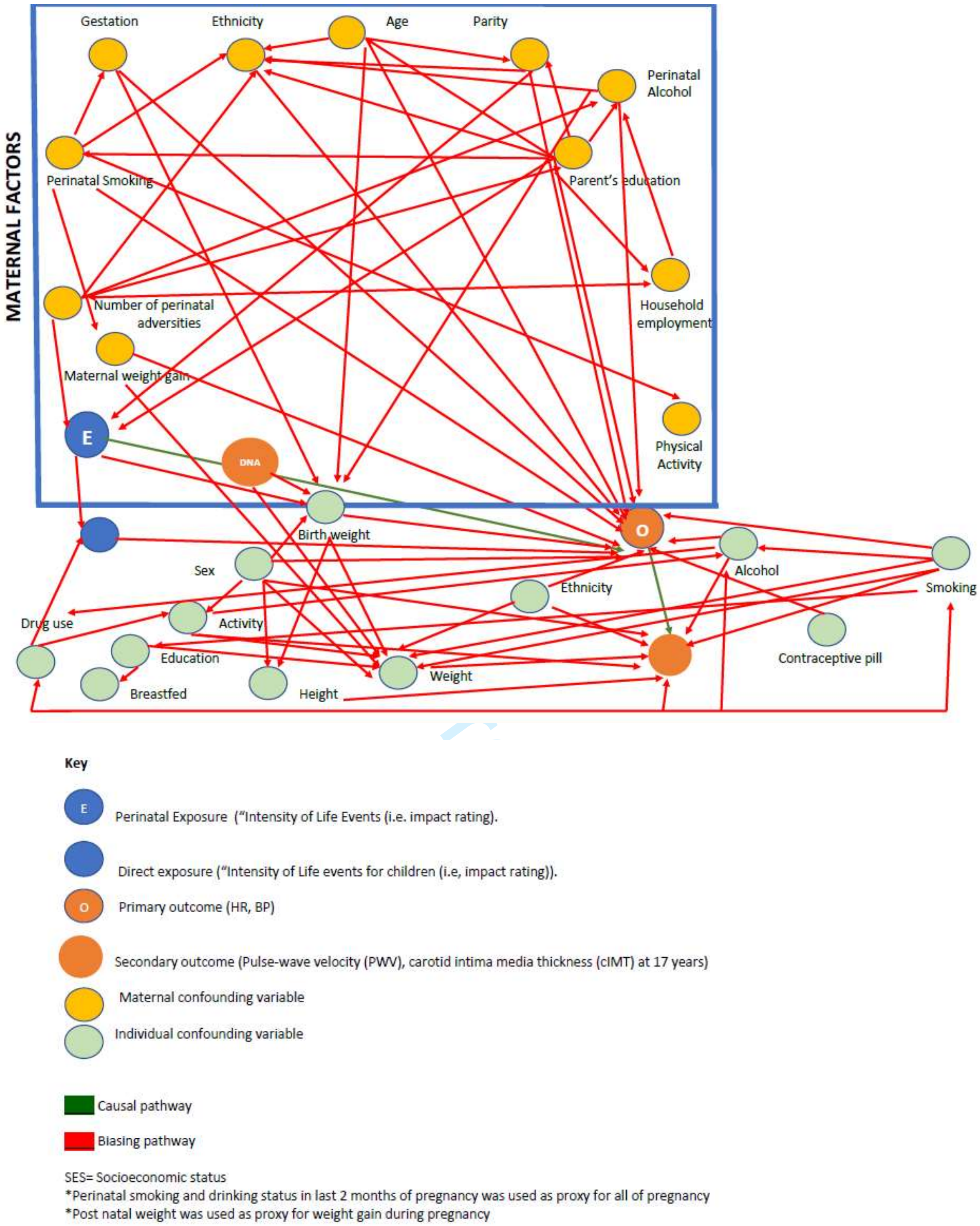


**Supplementary Figure S2:** Directed Acyclic Graph at seventeen years of age

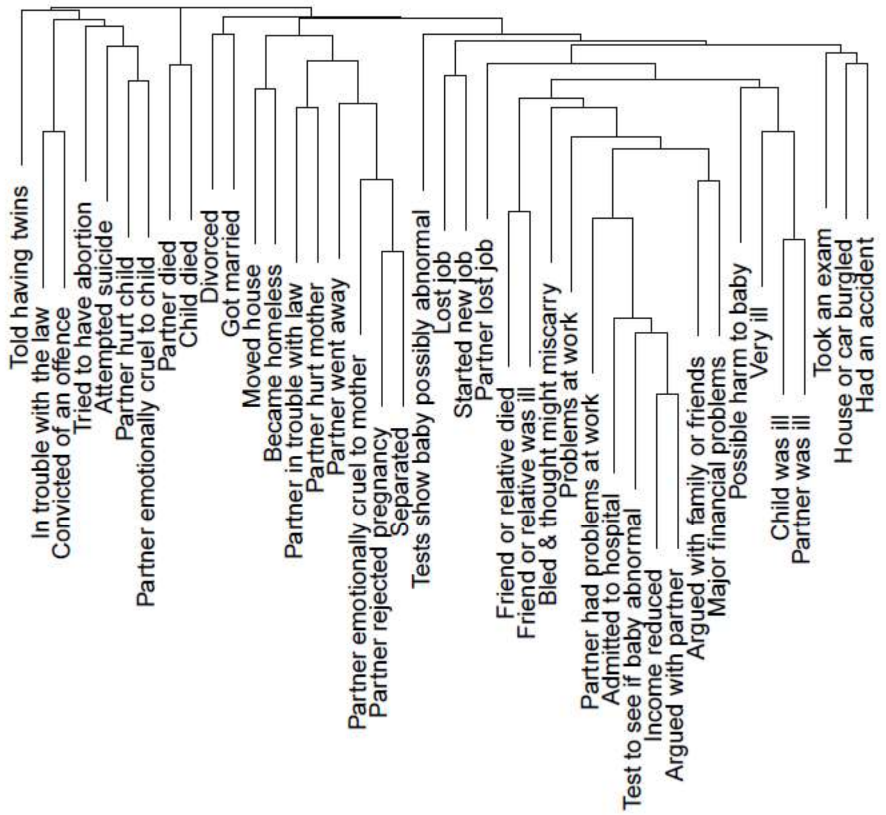


**Supplementary Figure S3:** Dendrogram of specific life events clusters

# Supplementary Table S1: Number of people reporting trauma and the perceived impact

| *Adversity event* | n | Occurred n(%) | Did not occur (n%) |
|---|---|---|---|
| Partner died | 12240 | 30 (0.2) | 12210 (99.8) |
| Child died | 12245 | 17 (0.1) | 12228 (99.9) |
| Friend or relative died | 12250 | 2588 (21.1) | 9662 (78.9) |
| Child was ill | 12238 | 2835 (23.2) | 9403 (76.8) |
| Partner was ill | 12240 | 2234 (18.3) | 10006 (81.7) |
| Friend or relative was ill | 12244 | 3182 (26) | 9062 (74) |
| Admitted to hospital | 12232 | 5508 (45) | 6724 (55) |
| In trouble with the law | 12246 | 138 (1.1) | 12108 (98.9) |
| Divorced | 12242 | 157 (1.3) | 12085 (98.7) |
| Partner rejected pregnancy | 12234 | 486 (4) | 11748 (96) |
| Very ill | 12244 | 1393 (11.4) | 10851 (88.6) |
| Partner lost job | 12225 | 1298 (10.6) | 10927 (89.4) |
| Partner had problems at work | 12223 | 3477 (28.4) | 8746 (71.6) |
| Problems at work | 12231 | 1927 (15.8) | 10304 (84.2) |
| Lost job | 12231 | 692 (5.7) | 11539 (94.3) |
| Partner went away | 12224 | 1534 (12.5) | 10690 (87.5) |
| Partner in trouble with law | 12228 | 415 (3.4) | 11813 (96.6) |
| Separated | 12232 | 665 (5.4) | 11567 (94.6) |
| Income reduced | 12237 | 6188 (50.6) | 6049 (49.4) |
| Argued with partner | 12243 | 7727 (63.1) | 4516 (36.9) |
| Argued with family or friends | 12243 | 2965 (24.2) | 9278 (75.8) |
| Moved house | 12243 | 2062 (16.8) | 10181 (83.2) |
| Partner hurt mother | 12230 | 321 (2.6) | 11909 (97.4) |
| Became homeless | 12238 | 239 (2) | 11999 (98) |
| Major financial problems | 12237 | 2319 (19) | 9918 (81) |
| Got married | 12236 | 492 (4) | 11744 (96) |
| Partner hurt child | 12229 | 31 (0.3) | 12198 (99.7) |
| Attempted suicide | 12237 | 28 (0.2) | 12209 (99.8) |
| Convicted of an offence | 12231 | 46 (0.4) | 12185 (99.6) |
| Bled & thought might miscarry | 12241 | 2068 (16.9) | 10173 (83.1) |
| Started new job | 12231 | 628 (5.1) | 11603 (94.9) |
| Test to see if baby abnormal | 12233 | 6430 (52.6) | 5803 (47.4) |
| Tests show baby possibly abnormal | 12230 | 549 (4.5) | 11681 (95.5) |
| Told having twins | 12239 | 113 (0.9) | 12126 (99.1) |
| Possible harm to baby | 12239 | 1287 (10.5) | 10952 (89.5) |
| Tried to have abortion | 12241 | 114 (0.9) | 12127 (99.1) |
| Took an exam | 12227 | 969 (7.9) | 11258 (92.1) |

For peer review only

| | | | |
|---|---|---|---|
| Partner emotionally cruel to mother | 12220 | 1100 (9) | 11120 (91) |
| Partner emotionally cruel to child | 12222 | 157 (1.3) | 12065 (98.7) |
| House or car burgled | 12239 | 1221 (10) | 11018 (90) |
| Had an accident | 12237 | 747 (6.1) | 11490 (93.9) |

For peer review only

STROBE Statement—checklist of items that should be included in reports of observational studies

| | Item No | Recommendation | Page No |
|---|---|---|---|
| **Title and abstract** | 1 | (*a*) Indicate the study's design with a commonly used term in the title or the abstract | 2 |
| | | (*b*) Provide in the abstract an informative and balanced summary of what was done and what was found | 2 |
| **Introduction** | | | |
| Background/rationale | 2 | Explain the scientific background and rationale for the investigation being reported | 4-5 |
| Objectives | 3 | State specific objectives, including any prespecified hypotheses | 5 |
| **Methods** | | | |
| Study design | 4 | Present key elements of study design early in the paper | 6-9 |
| Setting | 5 | Describe the setting, locations, and relevant dates, including periods of recruitment, exposure, follow-up, and data collection | 6-8 |
| Participants | 6 | (*a*) *Cohort study*—Give the eligibility criteria, and the sources and methods of selection of participants. Describe methods of follow-up<br>*Case-control study*—Give the eligibility criteria, and the sources and methods of case ascertainment and control selection. Give the rationale for the choice of cases and controls<br>*Cross-sectional study*—Give the eligibility criteria, and the sources and methods of selection of participants | 6 |
| | | (*b*) *Cohort study*—For matched studies, give matching criteria and number of exposed and unexposed<br>*Case-control study*—For matched studies, give matching criteria and the number of controls per case | N/A |
| Variables | 7 | Clearly define all outcomes, exposures, predictors, potential confounders, and effect modifiers. Give diagnostic criteria, if applicable | 6-8 |
| Data sources/ measurement | 8* | For each variable of interest, give sources of data and details of methods of assessment (measurement). Describe comparability of assessment methods if there is more than one group | 6-8 |
| Bias | 9 | Describe any efforts to address potential sources of bias | 15 |
| Study size | 10 | Explain how the study size was arrived at | 6 |
| Quantitative variables | 11 | Explain how quantitative variables were handled in the analyses. If applicable, describe which groupings were chosen and why | 8 |
| Statistical methods | 12 | (*a*) Describe all statistical methods, including those used to control for confounding | 8 |
| | | (*b*) Describe any methods used to examine subgroups and interactions | 8 |
| | | (*c*) Explain how missing data were addressed | 6 |
| | | (*d*) *Cohort study*—If applicable, explain how loss to follow-up was addressed<br>*Case-control study*—If applicable, explain how matching of cases and controls was addressed<br>*Cross-sectional study*—If applicable, describe analytical methods taking account of sampling strategy | 6 |
| | | (*e*) Describe any sensitivity analyses | N/A |

Continued on next page



For peer review only - http://bmjopen.bmj.com/site/about/guidelines.xhtml

| | | | |
|---|---|---|---|
| **Results** | | | |
| Participants | 13* | (a) Report numbers of individuals at each stage of study—eg numbers potentially eligible, examined for eligibility, confirmed eligible, included in the study, completing follow-up, and analysed | 6 |
| | | (b) Give reasons for non-participation at each stage | 6 |
| | | (c) Consider use of a flow diagram | - |
| Descriptive data | 14* | (a) Give characteristics of study participants (eg demographic, clinical, social) and information on exposures and potential confounders | 19 |
| | | (b) Indicate number of participants with missing data for each variable of interest | Tab 1 |
| | | (c) *Cohort study*—Summarise follow-up time (eg, average and total amount) | |
| Outcome data | 15* | *Cohort study*—Report numbers of outcome events or summary measures over time | Tab 1 |
| | | *Case-control study*—Report numbers in each exposure category, or summary measures of exposure | - |
| | | *Cross-sectional study*—Report numbers of outcome events or summary measures | - |
| Main results | 16 | (*a*) Give unadjusted estimates and, if applicable, confounder-adjusted estimates and their precision (eg, 95% confidence interval). Make clear which confounders were adjusted for and why they were included | 8 |
| | | (*b*) Report category boundaries when continuous variables were categorized | - |
| | | (*c*) If relevant, consider translating estimates of relative risk into absolute risk for a meaningful time period | - |
| Other analyses | 17 | Report other analyses done—eg analyses of subgroups and interactions, and sensitivity analyses | 8 |
| **Discussion** | | | |
| Key results | 18 | Summarise key results with reference to study objectives | 13 |
| Limitations | 19 | Discuss limitations of the study, taking into account sources of potential bias or imprecision. Discuss both direction and magnitude of any potential bias | 15 |
| Interpretation | 20 | Give a cautious overall interpretation of results considering objectives, limitations, multiplicity of analyses, results from similar studies, and other relevant evidence | 15 |
| Generalisability | 21 | Discuss the generalisability (external validity) of the study results | 15 |
| **Other information** | | | |
| Funding | 22 | Give the source of funding and the role of the funders for the present study and, if applicable, for the original study on which the present article is based | 16 |

*Give information separately for cases and controls in case-control studies and, if applicable, for exposed and unexposed groups in cohort and cross-sectional studies.

**Note:** An Explanation and Elaboration article discusses each checklist item and gives methodological background and published examples of transparent reporting. The STROBE checklist is best used in conjunction with this article (freely available on the Web sites of PLoS Medicine at http://www.plosmedicine.org/, Annals of Internal Medicine at http://www.annals.org/, and Epidemiology at http://www.epidem.com/). Information on the STROBE Initiative is available at www.strobe-statement.org.

For peer review only
